# Supplementary material for: A multivariate genome-wide association study of psycho-cardiometabolic multimorbidity
Source: PLoS Genet. 2023 Jun 30;19(6):e1010508. doi: 10.1371/journal.pgen.1010508 (PMC10343069; doi:10.1371/journal.pgen.1010508)
Supplement: S1 Appendix — (PDF) [file pgen.1010508.s015.pdf]

Supplementary Appendix

Table of Contents

Supplementary Methods....3

1 GWAS selection....3

2 LDSC regression quality control steps....3

3 Genomic SEM....3

4 Gene mapping....3

5 Positional mapping and pathway enrichment analysis....4

6 Polygenic risk score calculation and constructing psycho-cardiometabolic multimorbidity phenotype in UK Biobank....4

7 Genetic correlations....6

8 Mendelian randomization....7

Supplementary Results....8

1 Multivariate GWAS without UK Biobank....8

2 Mendelian randomization....8

Supplementary References....10

Supplementary Figures....15

Fig A. A flowchart of the analysis....15

Fig B. A quantile-quantile plot of the multivariate GWAS of psycho-cardiometabolic multimorbidity....16

Fig C. A quantile-quantile plot of heterogeneity Q_{SNP} *P*-values for the multivariate GWAS of psycho-cardiometabolic multimorbidity....17

Fig D. Enrichment in differentially expressed gene sets based on GTEx v8 30 general tissue types....18

Fig E. Enrichment in differentially expressed gene sets based on GTEx v8 54 tissue types....19

Fig F. MAGMA Tissue Expression Analysis in GTEx v8 53 tissue types....20

Fig G. MAGMA Tissue Expression Analysis in GTEx v8 30 general tissue types....21

Fig H. Causal effect of body mass index on multimorbidity....22

Fig I. Causal effect of body fat percentage on multimorbidity....23

Fig J. Causal effect of LDL cholesterol on multimorbidity....24

Fig K. Causal effect of total cholesterol on multimorbidity....25

Fig L. Causal effect of fasting insulin on multimorbidity. 26

Fig M. Causal effect of income on multimorbidity. 27

Fig N. Causal effect of insomnia on multimorbidity. 28

Fig O. Causal effect of childhood maltreatment on multimorbidity. 29

Fig P. Causal effect of neuroticism on multimorbidity. 30

Fig Q. Causal effect of diastolic blood pressure on multimorbidity. 31

Fig R. Causal effect of systolic blood pressure on multimorbidity. 32

Fig S. Causal effect of triglycerides on multimorbidity. 33

Fig T. Causal effect of intelligence on multimorbidity. 34

Fig U. Causal effect of worry on multimorbidity. 35

Fig V. Causal effect of SESA on multimorbidity. 36

Fig W. Causal effect of HDL cholesterol on multimorbidity. 37

Fig X. Causal effect of smoking status (current) on multimorbidity. 38

Fig Y. Causal effect of c-reactive protein on multimorbidity. 39

Supplementary Methods

1 GWAS selection

Data for major depression were obtained from a meta-analysis by Howard et al.[1] (n = 807,553), which was based on lifetime diagnoses of major depressive disorder (MDD) from clinical interviews, electronical medical records, self-report of symptoms or of help-seeking for depression and self-reported clinical diagnosis or treatment by a medical professional. Summary statistics for T2D and CAD were acquired from case-control meta-analyses by Mahajan et al.[2] (n = 898,130) and Nikpay et al.[3] (n = 184,305), respectively.

2 LDSC regression quality control steps

As part of the package, quality control (QC) steps included restricting genetic variants to HapMap 3 SNPs, filtering for single nucleotide polymorphisms (SNPs) with an imputation INFO score > 0.9 and minor allele frequency (MAF) > 0.01, as well as using precomputed European LD scores from the 1000 Genomes project (i.e., 'eur_w_ld_chr/'). INFO filtering was only performed for CAD, as INFO scores were not available for depression and T2D.

3 Genomic SEM

All default settings were kept, with the exception of 'genomic control', which was set to 'none' as the input univariate GWASs did not appear to suffer from genomic inflation related to population stratification. To help with model convergence, the residual variances were constrained to be positive for CAD and T2D. Once summary statistics for multimorbidity were obtained, the effective sample size (N_{eff}) was estimated following the approach described by Mallard et al.[4]. All of the above analyses were conducted using R version 4.0.0 [5].

4 Gene mapping

We broadened the genomic loci for annotation to include all known variants that are available in the 1000G reference panel and are in LD ($r^2 \geq 0.6$) with one of the non-heterogeneous, independent significant SNPs. Each genomic locus could therefore contain a mixture of independent significant SNPs and SNPs in LD with them (i.e., candidate SNPs). All

candidate SNPs were functionally annotated by performing ANNOVAR [6] gene-based annotation using Ensembl genes.

5 Positional mapping and pathway enrichment analysis

Positional mapping was selected to map SNPs to genes based on their physical position on the genome using ANNOVAR [6] annotations with 10 kb windows. eQTL mapping was used to map SNPs to genes based on significant eQTL associations (i.e., where gene expression was associated with allelic variation at the SNP) [7] within 1 Mb distance using GTEx v8 [8], Blood eQTL browser [9], PsychENCODE eQTLs , and BRAINEAC [11] repositories. Chromatin interaction mapping was selected to map SNPs to genes based on chromatin interactions between multimorbidity-associated regions and gene regions (without a distance boundary). For chromatin interactions Hi-C [12] data for 21 tissue/cell types were used.

For pathway enrichment analysis, we looked at the enrichment of the prioritised genes in biological pathways and functional categories, and their overrepresentation in differentially expressed gene (DEG) sets (i.e., sets of genes that are either up-regulated or down-regulated in a specific tissue compared to other tissue types).

6 Polygenic risk score calculation and constructing psycho-cardiometabolic multimorbidity phenotype in UK Biobank

Prior to calculating polygenic risk scores in the UKBB, we performed QC steps on base and target data, following the steps outlined by Choi et al.[13] The UKBB is a large prospective cohort with over 500,000 participants aged 40-69 years when recruited in 2006-2010 [14]. Out of the total sample, we selected 306,734 individuals of European ancestry that, in addition to individual-level genetic data, had phenotypic information available on depression, CAD and T2D.

To construct the psycho-cardiometabolic multimorbidity phenotype in UK Biobank, we first defined cases for single phenotypes: depression, coronary artery disease and type 2 diabetes based on information available at all time points (i.e., baseline, instance 1, 2 and 3).

6.1 Depression phenotype

Depression phenotype was derived using the UKBB field IDs specified in Glanville et al.[15] Briefly, cases were defined based on the following criteria:

1. Self-reported depression
   - Individuals who self-reported experiencing depression.
2. Lifetime depression - Mental Health Questionnaire (MHQ)
   - Individuals who met criteria for a lifetime history of depression, as defined in Glanville et al.[15] and Davis, et al.[16]. Example items included: “Have you ever had a time in your life when you felt sad, blue, or depressed for two weeks or more in a row?” or “Have you ever had a time in your life lasting two weeks or more when you lost interest in most things like hobbies, work, or activities that usually give you pleasure?”.
3. Depression (Smith) [17]
   - Individuals with a (1) single episode of probable major depression, (2) probable recurrent major depression (moderate), and (3) probable recurrent major depression (severe).
4. Hospital (ICD-10)
   - Individuals with either a primary or a secondary diagnosis of depressive episode (F32-F32.9) or recurrent depressive disorder (F33-F33.9).

6.2 Coronary artery disease

Cases were defined based on self-reported heart attack/angina (Data-Field 20002; value 1075 / 1074), or vascular heart problems diagnosed by a doctor (Data-Field 6150), specifically heart attack and angina.

6.3 Type 2 diabetes

Cases were defined based on self-reported type 2 diabetes (Data-Field 20002; value 1223) and doctor’s diagnosis of diabetes (Data-Field 2443).

6.4 Psycho-cardiometabolic multimorbidity

Psycho-cardiometabolic multimorbidity was defined as an ordinal variable, where:

- 0 = no disease
- 1 = any one disease (depression **or** CAD **or** T2D)
- 2 = any two diseases (depression and CAD **or** depression and T2D **or** CAD and T2D)
- 3 = all three diseases (depression and CAD and T2D)

PRS analysis was then performed using PRSice-2 [18], which uses linkage disequilibrium clumping and *P*-value thresholding method. The best model was derived by testing the inclusion of a variable number of SNPs (20-92,657 SNPs) using a range of *P*-value thresholds (P = 5e-8 to 1). Parameters that resulted in the largest R^2 (from a linear regression of the phenotype by the PRS) were used to generate PRS in the UKBB cohort. Note that while in this step the best model was chosen using a linear regression, the association of PRS with phenotypic multimorbidity in the target cohort was tested using multinomial logistic regression. This was done to obtain estimates of association for every possible combination of the three diseases: depression only; CAD only; T2D only; depression with CAD; depression with T2D; CAD with T2D; depression, CAD and T2D. Individuals with no disease were used as the reference category.

7 Genetic correlations

To explore genetic correlations with multimorbidity, we selected 18 traits that are commonly considered risk factors for either one, two or all three diseases (depression, CAD and T2D). The complete list included: insomnia [19], intelligence [20], childhood maltreatment [21], neuroticism [22], sensitivity to environmental stress and adversity (SESA) [23], worry [22], income (MTAG version) [24], low-density lipoprotein (LDL) cholesterol, high-density lipoprotein (HDL) cholesterol, total cholesterol, triglycerides [25], and seven traits obtained from the MRC IEU OpenGWAS database [26], which included current smoking (GWAS ID: ukb-a-225), BMI (GWAS ID: ukb-b-19953), body fat percentage (GWAS ID: ukb-b-8909), systolic blood pressure (GWAS ID: ieu-b-38), diastolic blood pressure (GWAS ID: ieu-b-39), C-reactive protein (UKBB GWAS: ukb-d-30710_irnt) and fasting insulin (UKBB GWAS: ieu-b-116).

8 Mendelian randomization

We conducted two-sample Mendelian randomization (MR) analysis [27] between 18 selected risk factors and multimorbidity using TwoSampleMR package[28] in R v4.0.0. The package harmonized exposure and outcome data and attempted to infer positive strand alleles using allele frequencies. Subsequently, independent SNPs with a GWAS $P < 5e-8$ were selected as instrumental variables for each exposure of interest. For childhood maltreatment, only 10 instruments were present using this threshold. As such, we decided to use a more lenient *p*-value threshold for this trait ($P < 5e-6$), as done elsewhere [29]. Independent SNPs were defined using 10,000 kb distance and $r^2 < 0.001$.

For each risk factor, we performed inverse-variance weighted (IVW) regression using multiplicative random effects, which combines SNP-exposure and SNP-outcome estimates to provide an overall estimate of the causal effect [27,30]. This method allows genetic instruments to be invalid instrumental variables if their pleiotropic effects are balanced. To assess the robustness of our IVW estimate, we conducted multiple sensitivity analyses using MR-Egger regression, MR-Egger intercept test, simple mode, weighted median, and weighted mode methods. The MR-Egger regression [31] relaxes the assumption that all genetic instruments are valid instrumental variables and allows a non-zero intercept. When the intercept differs from zero, it points to the potential presence of pleiotropy (a violation of the instrumental variable assumptions) and suggests that our primary IVW estimate may be biased. However, provided the InSIDE (Instrument Strength Independent of Direct Effect) assumption is satisfied, the MR-Egger regression provides a reliable estimate of the causal effect. The MR-Egger intercept test permits us to evaluate the presence of pleiotropic effects by assessing whether the intercept significantly differs from zero [32]. The simple mode [33], weighted median [34], and weighted mode [35] methods offer additional protection against potential biases due to horizontal pleiotropic effects or invalid instrumental variables that are not necessarily addressed by the IVW or MR-Egger analyses.

In addition to the above, we conducted leave-one-out analyses [36] which examined whether our findings are driven by any single SNP and performed MR Steiger test of directionality [37], which assessed whether the assumed causal direction between our exposure and outcome variables is valid.

Supplementary Results

1 Multivariate GWAS without UK Biobank

Overall, consistent findings were observed when repeating the analysis using the largest available GWASs for each trait, but this time excluding the UKBB cohort. Briefly, we observed positive genetic correlations among each pair of traits ($r_{g\ \mathrm{CAD\text{-}T2D}}$ = 0.41, *SE* = 0.04, *P* = 1e-20; $r_{g\ \mathrm{CAD\text{-}MD}}$ = 0.10, *SE* = 0.3, *P* = .002); $r_{g\ \mathrm{T2D\text{-}MD}}$ = 0.10, *SE* = 0.03, *P* = .002), which aligned closely in terms of their magnitude with the original genetic correlations with UKBB. The factor structure was also comparable, with the common factor explaining the greatest proportion of variance in T2D (R^2 = 0.42) and CAD (R^2 = 0.40), followed by depression (R^2 = 0.03). The multivariate GWAS identified 1,312 genome-wide significant SNPs, of which 30 were independent SNPs (S4 Table). Summary statistics for multimorbidity without UKBB correlated very strongly with summary statistics with UKBB (r_g = 0.98, *SE* = 0.01, *P* < .001). Eight of the 30 independent SNPs were a direct replication of the independent hits from the discovery multimorbidity GWAS, whereas the remaining three were suggestive of significance (all *P* < 5e-5). Using only non-heterogeneous SNPs as input to FUMA resulted in 1,196 mapped genes, six of which overlapped with genes identified in the discovery analysis using four different methods (MAGMA, positional, eQTL, and chromatin interaction mapping): *TMEM106B, RP11-145E5.5, C9orf53, CDKN2A, CDKN2B* and *TCF4.* For a full list of the genes see S9 Table.

2 Mendelian randomization

To assess the robustness of our Mendelian randomization results, we performed the MR-Egger intercept test, Steiger directionality test, and evaluated SNP heterogeneity statistic for each trait. The MR-Egger intercept only deviated from zero for total cholesterol (intercept: -0.005, *SE* = 0.002, *P* = 0.009) and HDL cholesterol (intercept: -0.005, *SE* = 0.002, *P* = 0.003). While there was significant heterogeneity among the instrumental variables (S13 Table) for most of the risk factors, leave-one-out analyses indicated that no single variant was driving the results (S7-S24 Fig). Lastly, Steiger analysis suggested that the direction of the causal effect between our exposure and outcome variables were correct (S14 Table).

MRlap analysis which corrects for sample overlap, weak instrument bias and winner's curse revealed directionally consistent observed and bias-corrected effects. In many cases, the bias-corrected estimates were significantly larger than the observed effects. None of the traits had smaller corrected effects than the observed ones (S13 Table). These results suggest that the average bias in our IVW estimates was towards the null.

Supplementary References

1. Howard DM, Adams MJ, Clarke T-K, Hafferty JD, Gibson J, Shirali M, et al. Genome-wide meta-analysis of depression identifies 102 independent variants and highlights the importance of the prefrontal brain regions. Nat Neurosci. 2019;22: 343–352. doi:10.1038/s41593-018-0326-7

2. Mahajan A, Taliun D, Thurner M, Robertson NR, Torres JM, Rayner NW, et al. Fine-mapping type 2 diabetes loci to single-variant resolution using high-density imputation and islet-specific epigenome maps. Nature Genetics. 2018;50: 1505–1513. doi:10.1038/s41588-018-0241-6

3. Nikpay M, Goel A, Won H-H, Hall LM, Willenborg C, Kanoni S, et al. A comprehensive 1000 Genomes-based genome-wide association meta-analysis of coronary artery disease. Nat Genet. 2015;47: 1121–1130. doi:10.1038/ng.3396

4. Mallard TT, Linnér RK, Grotzinger AD, Sanchez-Roige S, Seidlitz J, Okbay A, et al. Multivariate GWAS of psychiatric disorders and their cardinal symptoms reveal two dimensions of cross-cutting genetic liabilities. Genetics; 2019 Apr. doi:10.1101/603134

5. R Core Team. R: A language and environment for statistical computing. R Foundation for Statistical Computing, Vienna, Austria. URL https://www.R-project.org/. 2020. Available: https://www.R-project.org/

6. Wang K, Li M, Hakonarson H. ANNOVAR: functional annotation of genetic variants from high-throughput sequencing data. Nucleic Acids Research. 2010;38: e164–e164. doi:10.1093/nar/gkq603

7. Watanabe K, Taskesen E, van Bochoven A, Posthuma D. Functional mapping and annotation of genetic associations with FUMA. Nat Commun. 2017;8: 1826. doi:10.1038/s41467-017-01261-5

8. The GTEx Consortium. The Genotype-Tissue Expression (GTEx) pilot analysis: Multitissue gene regulation in humans. Science. 2015;348: 648–660. doi:10.1126/science.1262110

9. Westra H-J, Peters MJ, Esko T, Yaghootkar H, Schurmann C, Kettunen J, et al. Systematic identification of trans eQTLs as putative drivers of known disease associations. Nat Genet. 2013;45: 1238–1243. doi:10.1038/ng.2756

10. Wang D, Liu S, Warrell J, Won H, Shi X, Navarro FCP, et al. Comprehensive functional genomic resource and integrative model for the human brain. Science. 2018;362: eaat8464. doi:10.1126/science.aat8464

11. Ramasamy A, Trabzuni D, Guelfi S, Varghese V, Smith C, Walker R, et al. Genetic variability in the regulation of gene expression in ten regions of the human brain. Nat Neurosci. 2014;17: 1418–1428. doi:10.1038/nn.3801

12. Schmitt AD, Hu M, Jung I, Xu Z, Qiu Y, Tan CL, et al. A Compendium of Chromatin Contact Maps Reveals Spatially Active Regions in the Human Genome. Cell Reports. 2016;17: 2042–2059. doi:10.1016/j.celrep.2016.10.061

13. Choi SW, Mak TS-H, O'Reilly PF. Tutorial: a guide to performing polygenic risk score analyses. Nat Protoc. 2020;15: 2759–2772. doi:10.1038/s41596-020-0353-1

14. Sudlow C, Gallacher J, Allen N, Beral V, Burton P, Danesh J, et al. UK Biobank: An Open Access Resource for Identifying the Causes of a Wide Range of Complex Diseases of Middle and Old Age. PLoS Med. 2015;12: e1001779. doi:10.1371/journal.pmed.1001779

15. Glanville KP, Coleman JRI, Howard DM, Pain O, Hanscombe KB, Jermy B, et al. Multiple measures of depression to enhance validity of major depressive disorder in the UK Biobank. BJPsych open. 2021;7: e44. doi:10.1192/bjo.2020.145

16. Davis KAS, Coleman JRI, Adams M, Allen N, Breen G, Cullen B, et al. Mental health in UK Biobank – development, implementation and results from an online questionnaire completed by 157 366 participants: a reanalysis. BJPsych open. 2020;6: e18. doi:10.1192/bjo.2019.100

17. Smith DJ, Nicholl BI, Cullen B, Martin D, Ul-Haq Z, Evans J, et al. Prevalence and Characteristics of Probable Major Depression and Bipolar Disorder within UK Biobank: Cross-Sectional Study of 172,751 Participants. Potash JB, editor. PLoS ONE. 2013;8: e75362. doi:10.1371/journal.pone.0075362

18. Choi SW, O'Reilly PF. PRSice-2: Polygenic Risk Score software for biobank-scale data. GigaScience. 2019;8: giz082. doi:10.1093/gigascience/giz082

19. Jansen PR, Watanabe K, Stringer S, Skene N, Bryois J, Hammerschlag AR, et al. Genome-wide analysis of insomnia in 1,331,010 individuals identifies new risk loci and functional pathways. Nat Genet. 2019;51: 394–403. doi:10.1038/s41588-018-0333-3

20. Savage JE, Jansen PR, Stringer S, Watanabe K, Bryois J, de Leeuw CA, et al. Genome-wide association meta-analysis in 269,867 individuals identifies new genetic and functional links to intelligence. Nat Genet. 2018;50: 912–919. doi:10.1038/s41588-018-0152-6

21. Warrier V, Kwong ASF, Luo M, Dalvie S, Croft J, Sallis HM, et al. Gene–environment correlations and causal effects of childhood maltreatment on physical and mental health: a genetically informed approach. The Lancet Psychiatry. 2021; S2215036620305691. doi:10.1016/S2215-0366(20)30569-1

22. Nagel M, Jansen PR, Stringer S, Watanabe K, de Leeuw CA, Bryois J, et al. Meta-analysis of genome-wide association studies for neuroticism in 449,484 individuals identifies novel genetic loci and pathways. Nat Genet. 2018;50: 920–927. doi:10.1038/s41588-018-0151-7

23. Nagel M, Speed D, Sluis S, Østergaard SD. Genome-wide association study of the sensitivity to environmental stress and adversity neuroticism cluster. Acta Psychiatr Scand. 2020;141: 476–478. doi:10.1111/acps.13155

24. Hill WD, Davies NM, Ritchie SJ, Skene NG, Bryois J, Bell S, et al. Genome-wide analysis identifies molecular systems and 149 genetic loci associated with income. Nat Commun. 2019;10: 5741. doi:10.1038/s41467-019-13585-5

25. Willer CJ, Schmidt EM, Sengupta S, Peloso GM, Gustafsson S, Kanoni S, et al. Discovery and refinement of loci associated with lipid levels. Nat Genet. 2013;45: 1274–1283. doi:10.1038/ng.2797

26. Elsworth B, Lyon M, Alexander T, Liu Y, Matthews P, Hallett J, et al. The MRC IEU OpenGWAS data infrastructure. Genetics; 2020 Aug. doi:10.1101/2020.08.10.244293

27. Burgess S, Butterworth A, Thompson SG. Mendelian Randomization Analysis With Multiple Genetic Variants Using Summarized Data. Genet Epidemiol. 2013;37: 658–665. doi:10.1002/gepi.21758

28. Hemani G, Zheng J, Elsworth B, Wade KH, Haberland V, Baird D, et al. The MR-Base platform supports systematic causal inference across the human phenome. eLife. 2018;7: e34408. doi:10.7554/eLife.34408

29. Tylee DS, Lee YK, Wendt FR, Pathak GA, Levey DF, De Angelis F, et al. An Atlas of Genetic Correlations and Genetically Informed Associations Linking Psychiatric and Immune-Related Phenotypes. JAMA Psychiatry. 2022;79: 667. doi:10.1001/jamapsychiatry.2022.0914

30. Burgess S, Davey Smith G, Davies NM, Dudbridge F, Gill D, Glymour MM, et al. Guidelines for performing Mendelian randomization investigations. Wellcome Open Res. 2020;4: 186. doi:10.12688/wellcomeopenres.15555.2

31. Bowden J, Davey Smith G, Burgess S. Mendelian randomization with invalid instruments: effect estimation and bias detection through Egger regression. International Journal of Epidemiology. 2015;44: 512–525. doi:10.1093/ije/dyv080

32. Burgess S, Thompson SG. Interpreting findings from Mendelian randomization using the MR-Egger method. Eur J Epidemiol. 2017;32: 377–389. doi:10.1007/s10654-017-0255-x

33. Hartwig FP, Davey Smith G, Bowden J. Robust inference in summary data Mendelian randomization via the zero modal pleiotropy assumption. International Journal of Epidemiology. 2017;46: 1985–1998. doi:10.1093/ije/dyx102

34. Bowden J, Davey Smith G, Haycock PC, Burgess S. Consistent Estimation in Mendelian Randomization with Some Invalid Instruments Using a Weighted Median Estimator. Genet Epidemiol. 2016;40: 304–314. doi:10.1002/gepi.21965

35. Burgess S, Foley CN, Allara E, Staley JR, Howson JMM. A robust and efficient method for Mendelian randomization with hundreds of genetic variants. Nat Commun. 2020;11: 376. doi:10.1038/s41467-019-14156-4

36. Burgess S, Bowden J, Fall T, Ingelsson E, Thompson SG. Sensitivity Analyses for Robust Causal Inference from Mendelian Randomization Analyses with Multiple Genetic Variants. Epidemiology. 2017;28: 30–42. doi:10.1097/EDE.0000000000000559

37. Hemani G, Tilling K, Smith GD. Orienting the causal relationship between imprecisely measured traits using GWAS summary data. PLoS Genetics. 2017;13: e1007081. doi:10.1371/journal.pgen.1007081

Supplementary Figures

Fig A. A flowchart of the analysis.

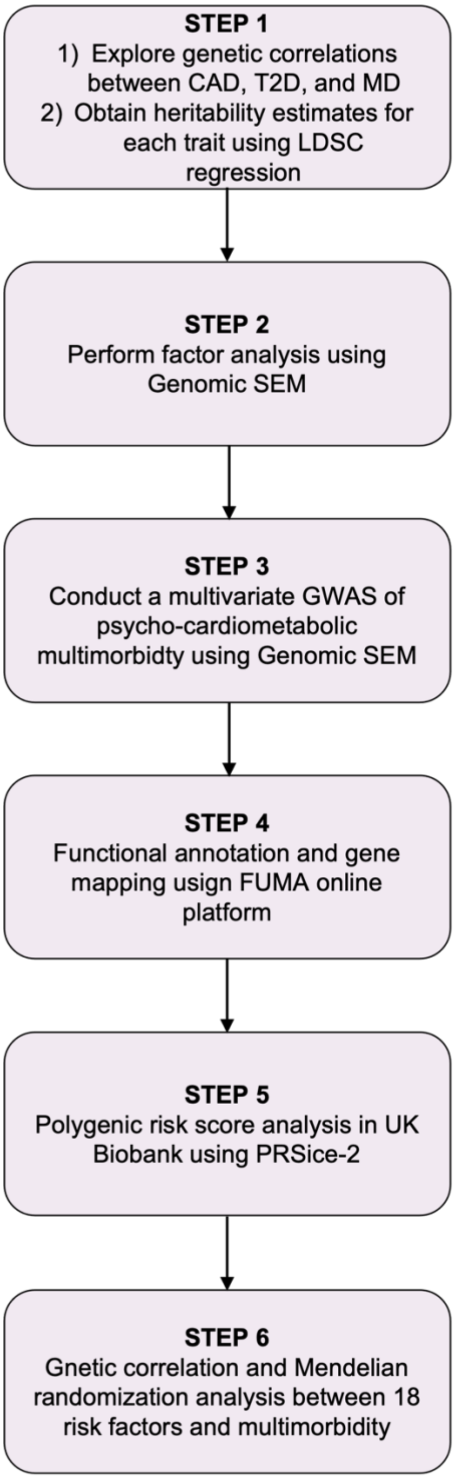

CAD, coronary artery disease; T2D, type 2 diabetes; MD, major depression; LDSC, linkage disequilibrium score regression; Genomic SEM, genomic structural equation modelling.

Fig B. A quantile-quantile plot of the multivariate GWAS of psycho-cardiometabolic multimorbidity.

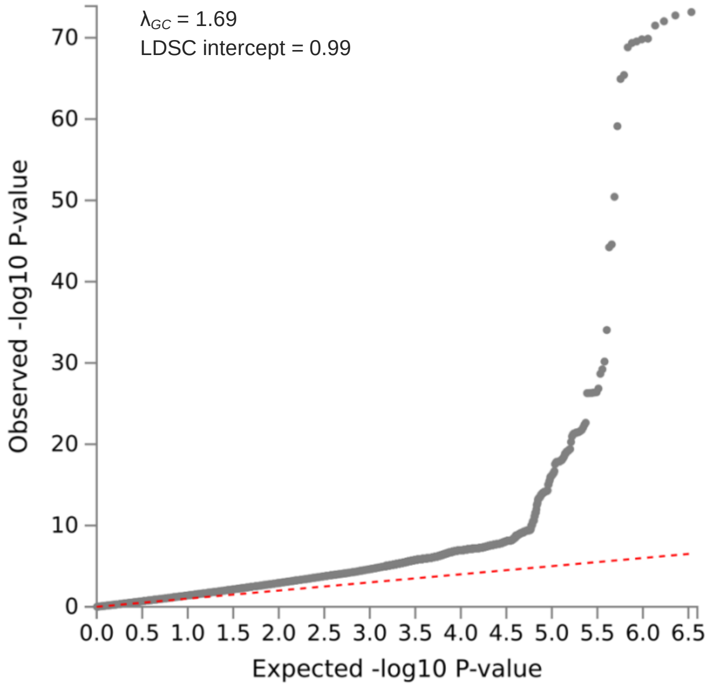

The observed –log10(*P*) -values in grey are plotted against their expected values under the null hypothesis (red line). LDSC, linkage disequilibrium score regression.

Fig C. A quantile-quantile plot of heterogeneity Q_{SNP} *P*-values for the multivariate GWAS of psycho-cardiometabolic multimorbidity.

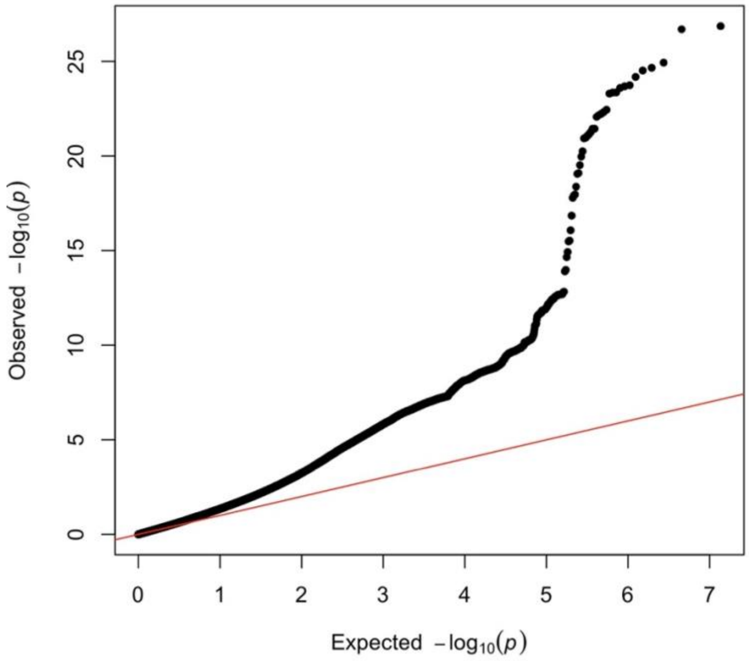

The observed –log10(*P*) -values in black are plotted against their expected values under the null hypothesis (red line). To assess a possible inflation of heterogeneity for multimorbidity-associated SNPs (rather than SNPs tagging one or two of the constituting diseases), variants with a heterogeneity Q_{SNP} $P < 5e\text{-}8$ and directionally discordant univariate effect estimates were removed prior to plotting this data. SNPs = single nucleotide polymorphisms.

Fig D. Enrichment in differentially expressed gene sets based on GTEx v8 30 general tissue types.

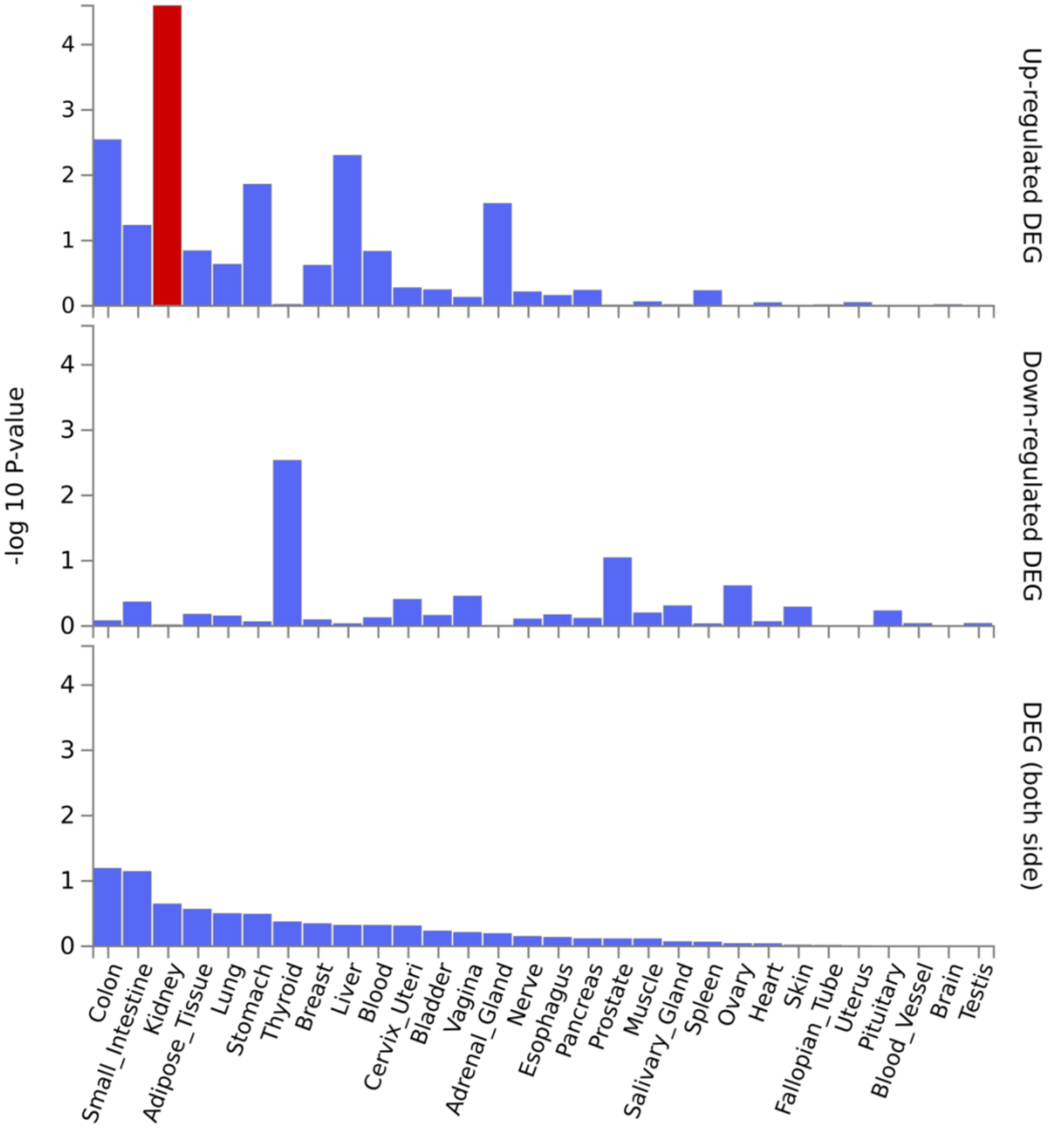

Significantly enriched differentially expressed gene (DEG) sets with a Bonferroni corrected *P*-value ≤ 0.05 and a log fold change ≥ 0.58 are highlighted in red.

Fig E. Enrichment in differentially expressed gene sets based on GTEx v8 54 tissue types.

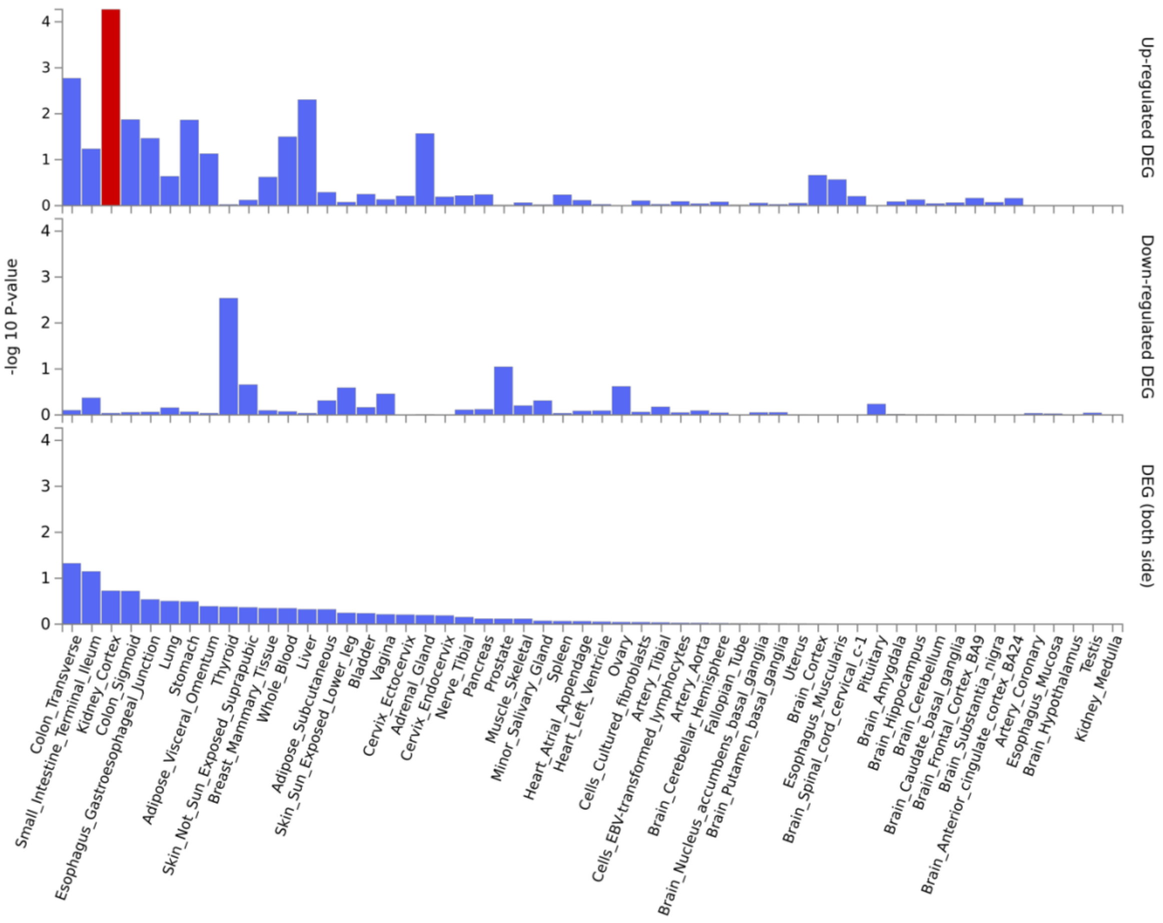

Significantly enriched differentially expressed gene (DEG) sets with a Bonferroni corrected *P*-value ≤ 0.05 and a log fold change ≥ 0.58 are highlighted in red.

Fig F. MAGMA Tissue Expression Analysis in GTEx v8 53 tissue types.

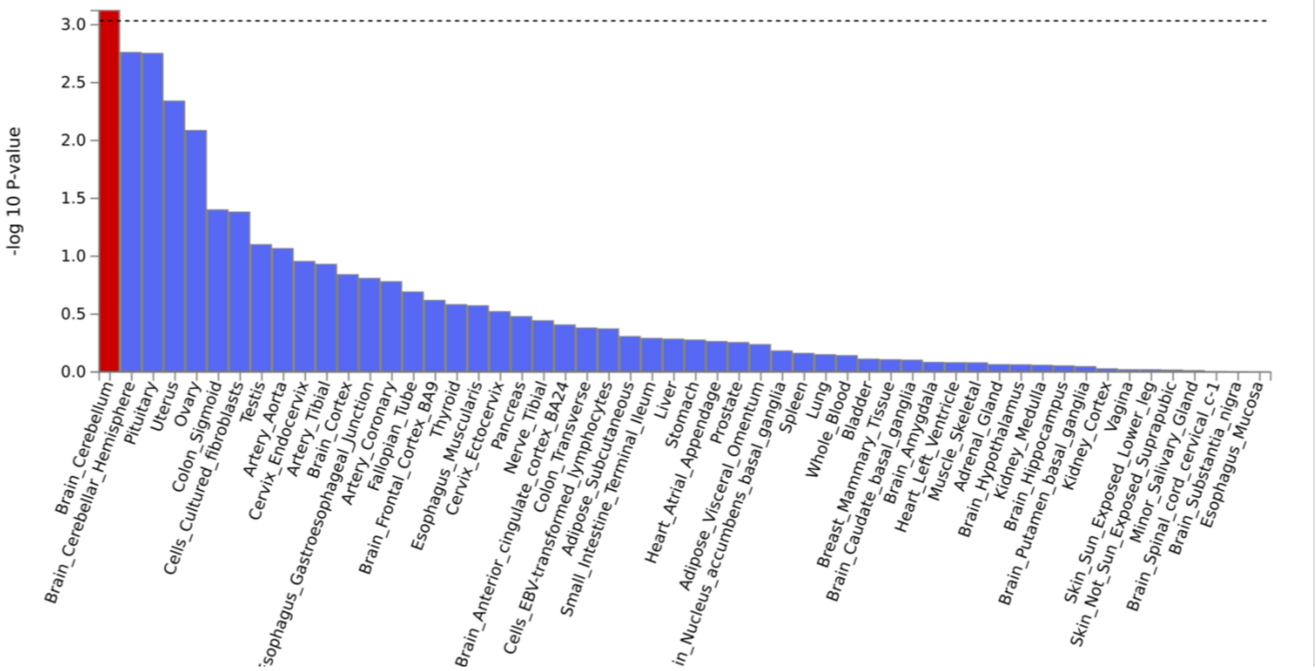

Using the full distribution of genome-wide association SNP *p*-values. SNP, single nucleotide polymorphism.

Fig G. MAGMA Tissue Expression Analysis in GTEx v8 30 general tissue types.

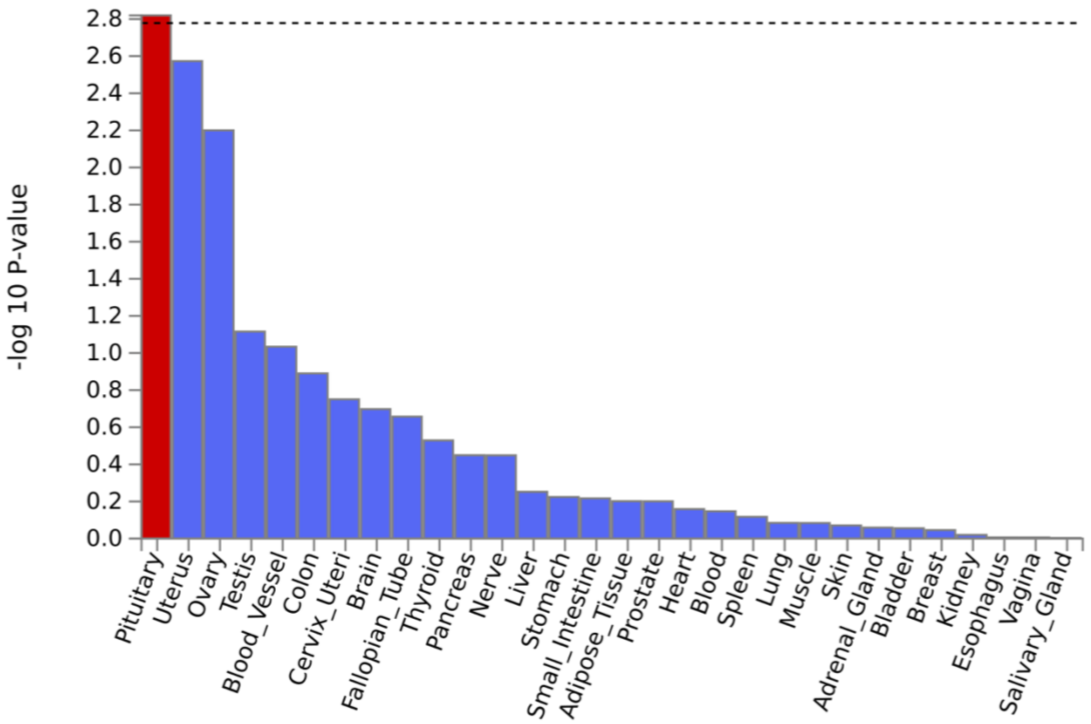

Using the full distribution of genome-wide association SNP *p*-values. SNP, single nucleotide polymorphism.

Fig H. Causal effect of body mass index on multimorbidity.

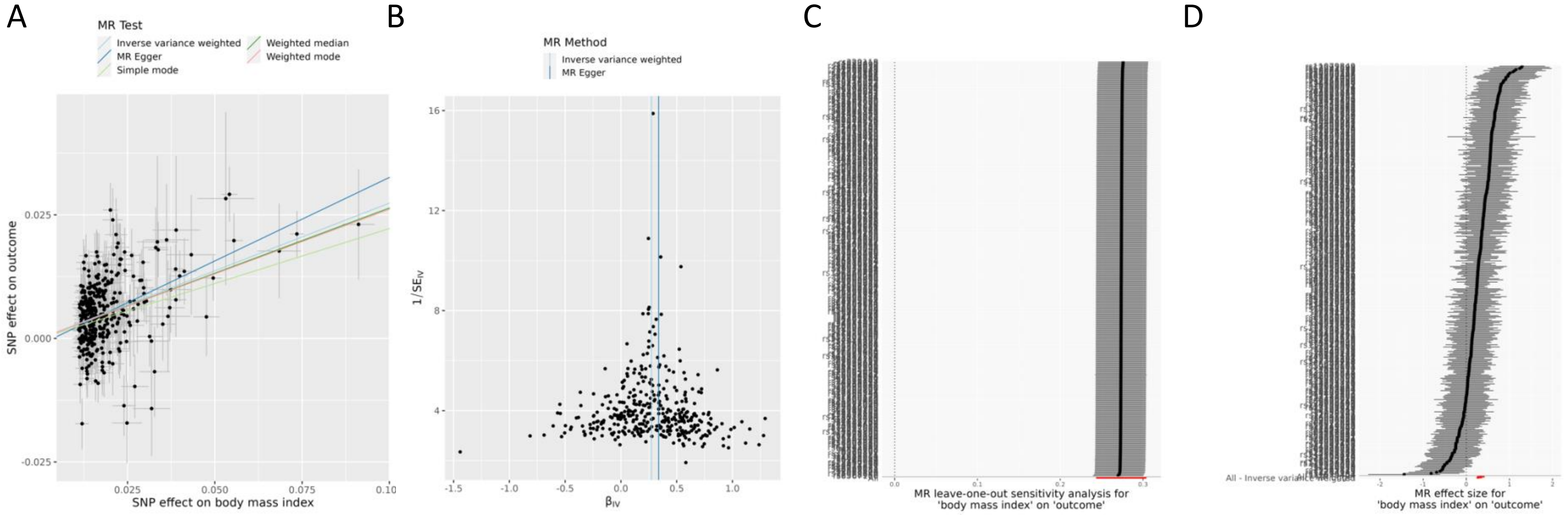

(A) A scatter plot showing SNP effects on the exposure and outcome. **(B)** A funnel plot of the association between exposure and outcome using each SNP individually against the inverse of the standard error of the causal effect. **(C)** Leave-one-out-analysis showing causal estimates after excluding each SNP from the analysis one-by-one. The IVW estimate using all SNPs is depicted in red. **(D)** Forest plot showing causal estimates using each SNP as a separate instrument (in black) and their combined effect using inverse-variance weighted and MR-Egger methods (in red).

Fig I. Causal effect of body fat percentage on multimorbidity.

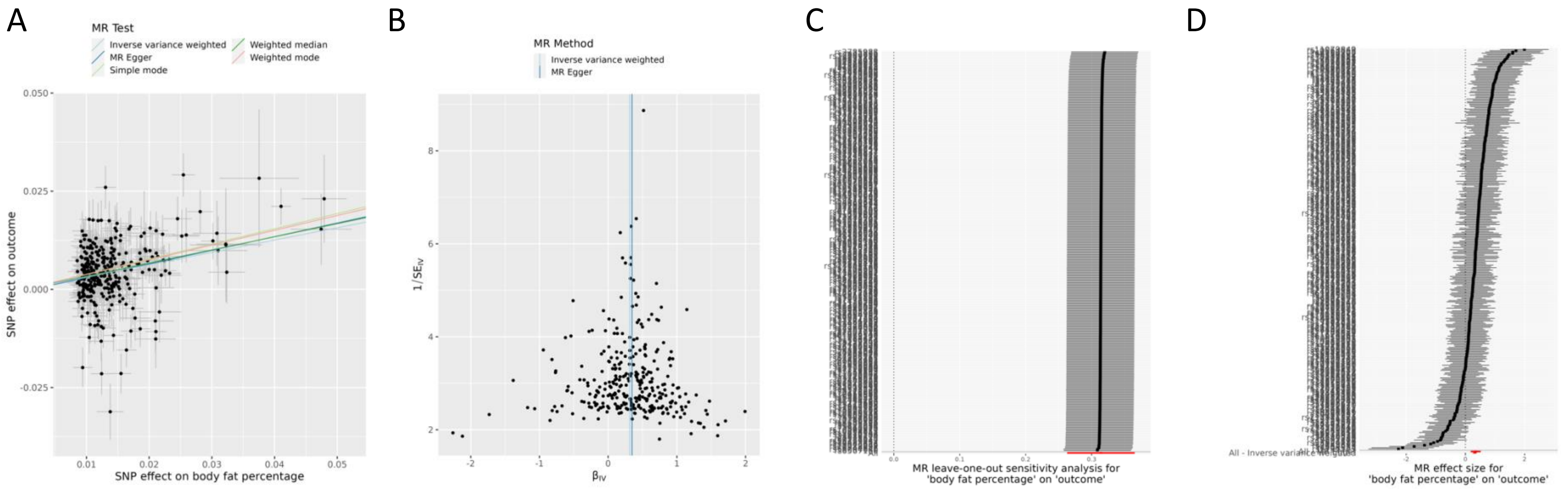

(A) A scatter plot showing SNP effects on the exposure and outcome. **(B)** A funnel plot of the association between exposure and outcome using each SNP individually against the inverse of the standard error of the causal effect. **(C)** Leave-one-out-analysis showing causal estimates after excluding each SNP from the analysis one-by-one. The IVW estimate using all SNPs is depicted in red. **(D)** Forest plot showing causal estimates using each SNP as a separate instrument (in black) and their combined effect using inverse-variance weighted and MR-Egger methods (in red).

Fig J. Causal effect of LDL cholesterol on multimorbidity.

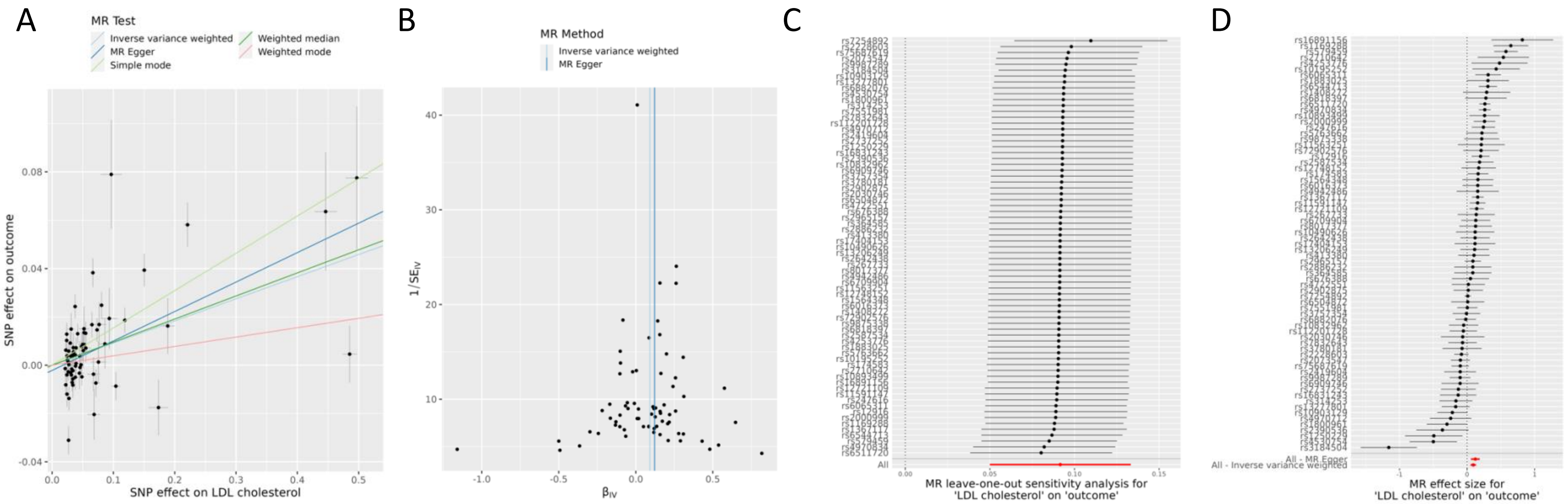

(A) A scatter plot showing SNP effects on the exposure and outcome. **(B)** A funnel plot of the association between exposure and outcome using each SNP individually against the inverse of the standard error of the causal effect. **(C)** Leave-one-out-analysis showing causal estimates after excluding each SNP from the analysis one-by-one. The IVW estimate using all SNPs is depicted in red. **(D)** Forest plot showing causal estimates using each SNP as a separate instrument (in black) and their combined effect using inverse-variance weighted and MR-Egger methods (in red). LDL, low-density lipoprotein.

Fig K. Causal effect of total cholesterol on multimorbidity.

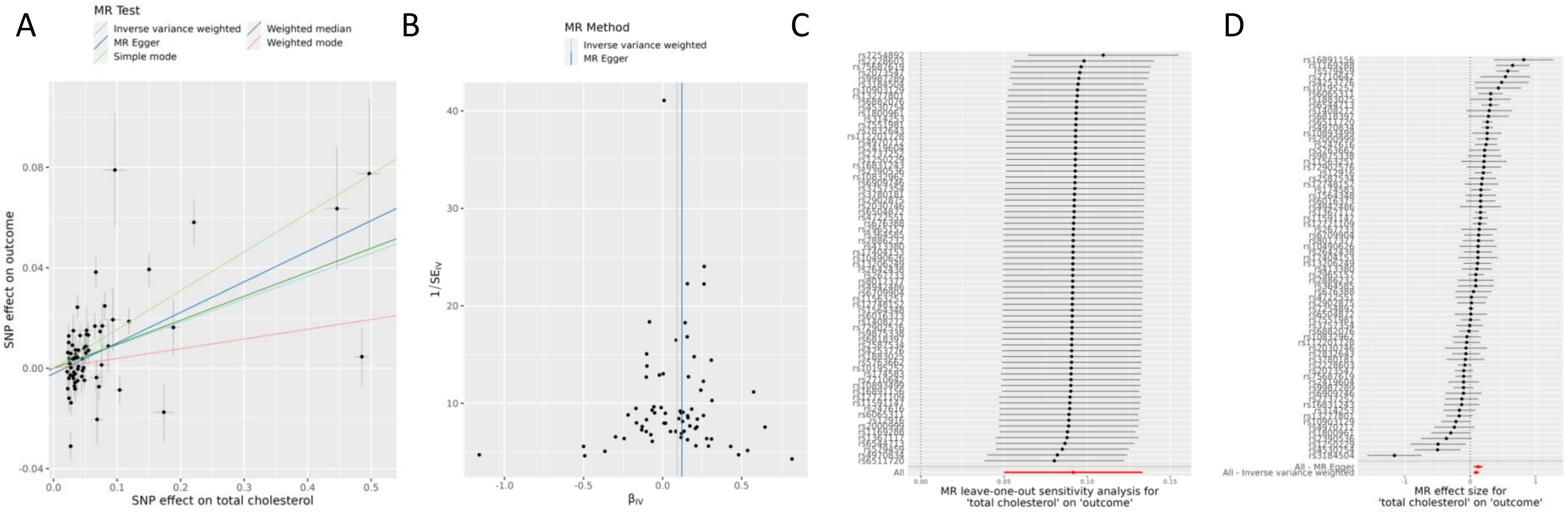

(A) A scatter plot showing SNP effects on the exposure and outcome. **(B)** A funnel plot of the association between exposure and outcome using each SNP individually against the inverse of the standard error of the causal effect. **(C)** Leave-one-out-analysis showing causal estimates after excluding each SNP from the analysis one-by-one. The IVW estimate using all SNPs is depicted in red. **(D)** Forest plot showing causal estimates using each SNP as a separate instrument (in black) and their combined effect using inverse-variance weighted and MR-Egger methods (in red).

Fig L. Causal effect of fasting insulin on multimorbidity.

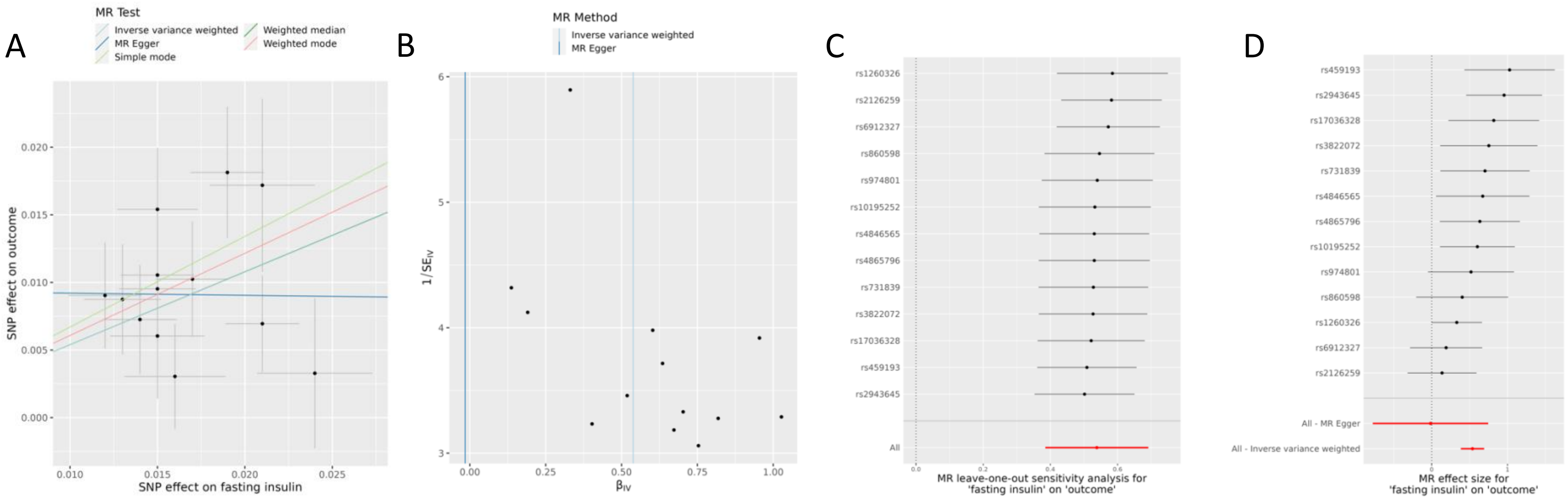

(A) A scatter plot showing SNP effects on the exposure and outcome. **(B)** A funnel plot of the association between exposure and outcome using each SNP individually against the inverse of the standard error of the causal effect. **(C)** Leave-one-out-analysis showing causal estimates after excluding each SNP from the analysis one-by-one. The IVW estimate using all SNPs is depicted in red. **(D)** Forest plot showing causal estimates using each SNP as a separate instrument (in black) and their combined effect using inverse-variance weighted and MR-Egger methods (in red).

Fig M. Causal effect of income on multimorbidity.

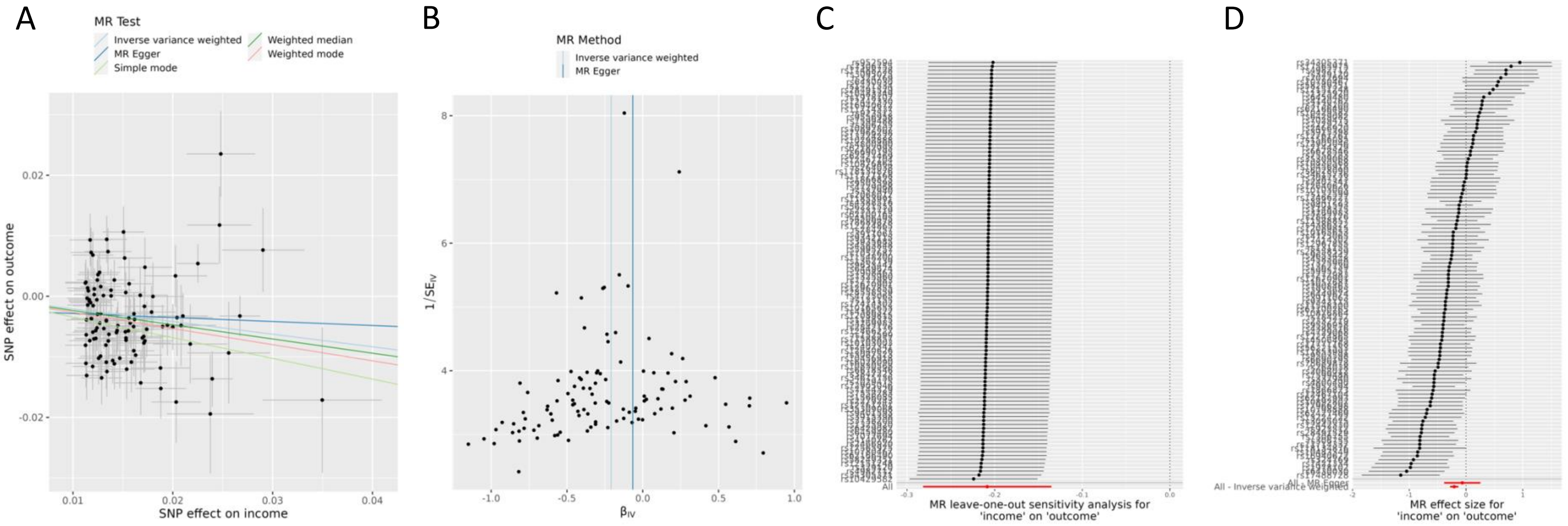

(A) A scatter plot showing SNP effects on the exposure and outcome. **(B)** A funnel plot of the association between exposure and outcome using each SNP individually against the inverse of the standard error of the causal effect. **(C)** Leave-one-out-analysis showing causal estimates after excluding each SNP from the analysis one-by-one. The IVW estimate using all SNPs is depicted in red. **(D)** Forest plot showing causal estimates using each SNP as a separate instrument (in black) and their combined effect using inverse-variance weighted and MR-Egger methods (in red).

Fig N. Causal effect of insomnia on multimorbidity.

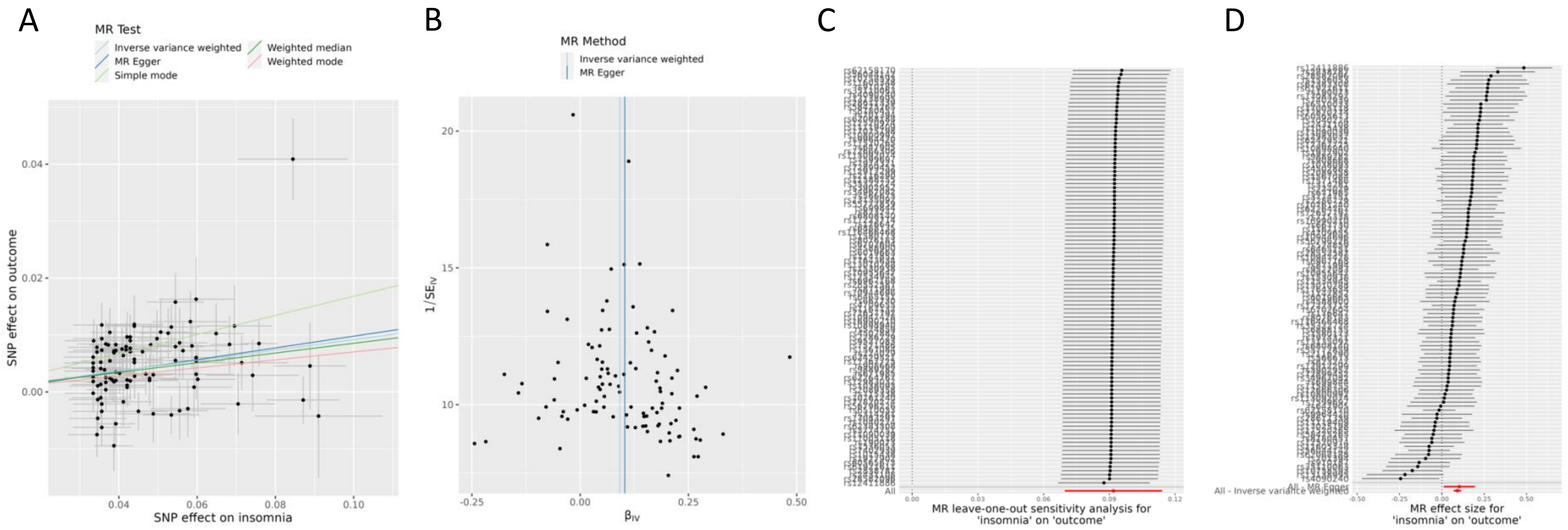

(A) A scatter plot showing SNP effects on the exposure and outcome. **(B)** A funnel plot of the association between exposure and outcome using each SNP individually against the inverse of the standard error of the causal effect. **(C)** Leave-one-out-analysis showing causal estimates after excluding each SNP from the analysis one-by-one. The IVW estimate using all SNPs is depicted in red. **(D)** Forest plot showing causal estimates using each SNP as a separate instrument (in black) and their combined effect using inverse-variance weighted and MR-Egger methods (in red).

Fig O. Causal effect of childhood maltreatment on multimorbidity.

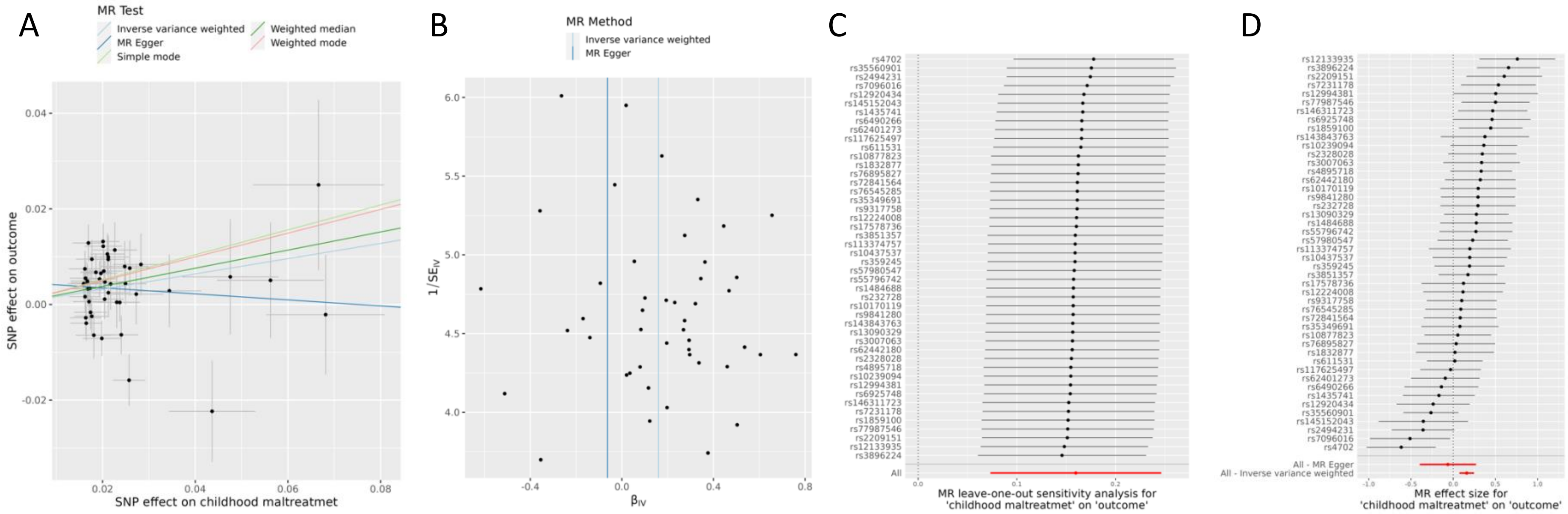

(A) A scatter plot showing SNP effects on the exposure and outcome. **(B)** A funnel plot of the association between exposure and outcome using each SNP individually against the inverse of the standard error of the causal effect. **(C)** Leave-one-out-analysis showing causal estimates after excluding each SNP from the analysis one-by-one. The IVW estimate using all SNPs is depicted in red. **(D)** Forest plot showing causal estimates using each SNP as a separate instrument (in black) and their combined effect using inverse-variance weighted and MR-Egger methods (in red).

Fig P. Causal effect of neuroticism on multimorbidity.

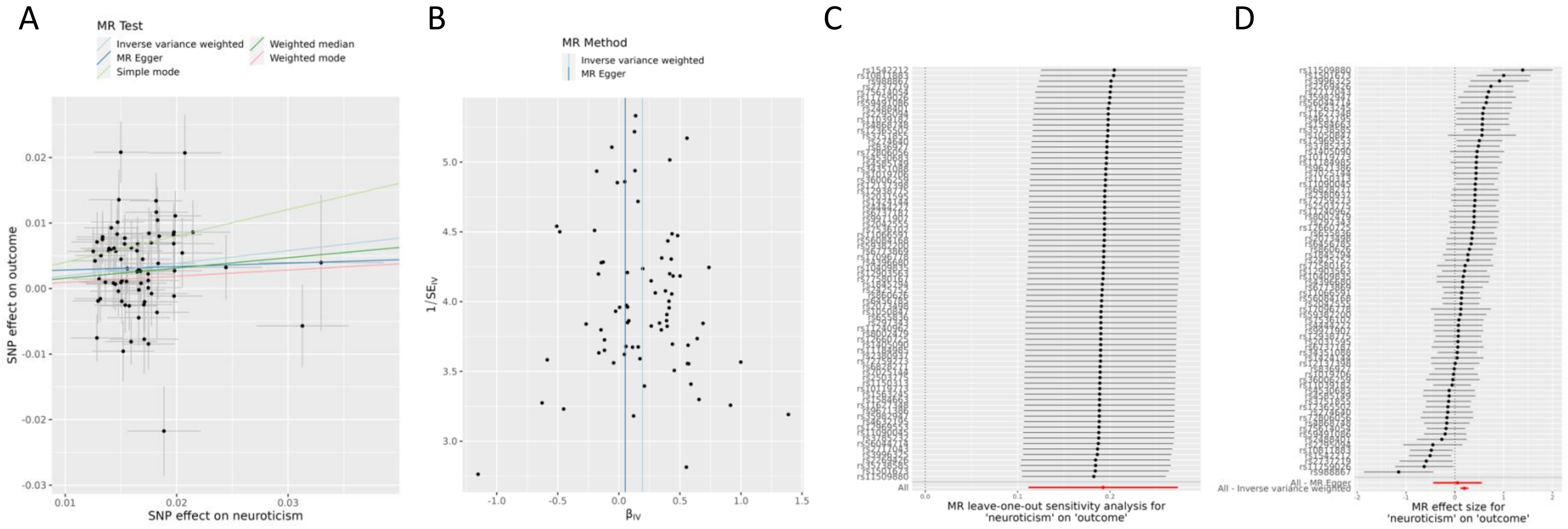

(A) A scatter plot showing SNP effects on the exposure and outcome. **(B)** A funnel plot of the association between exposure and outcome using each SNP individually against the inverse of the standard error of the causal effect. **(C)** Leave-one-out-analysis showing causal estimates after excluding each SNP from the analysis one-by-one. The IVW estimate using all SNPs is depicted in red. **(D)** Forest plot showing causal estimates using each SNP as a separate instrument (in black) and their combined effect using inverse-variance weighted and MR-Egger methods (in red).

Fig Q. Causal effect of diastolic blood pressure on multimorbidity.

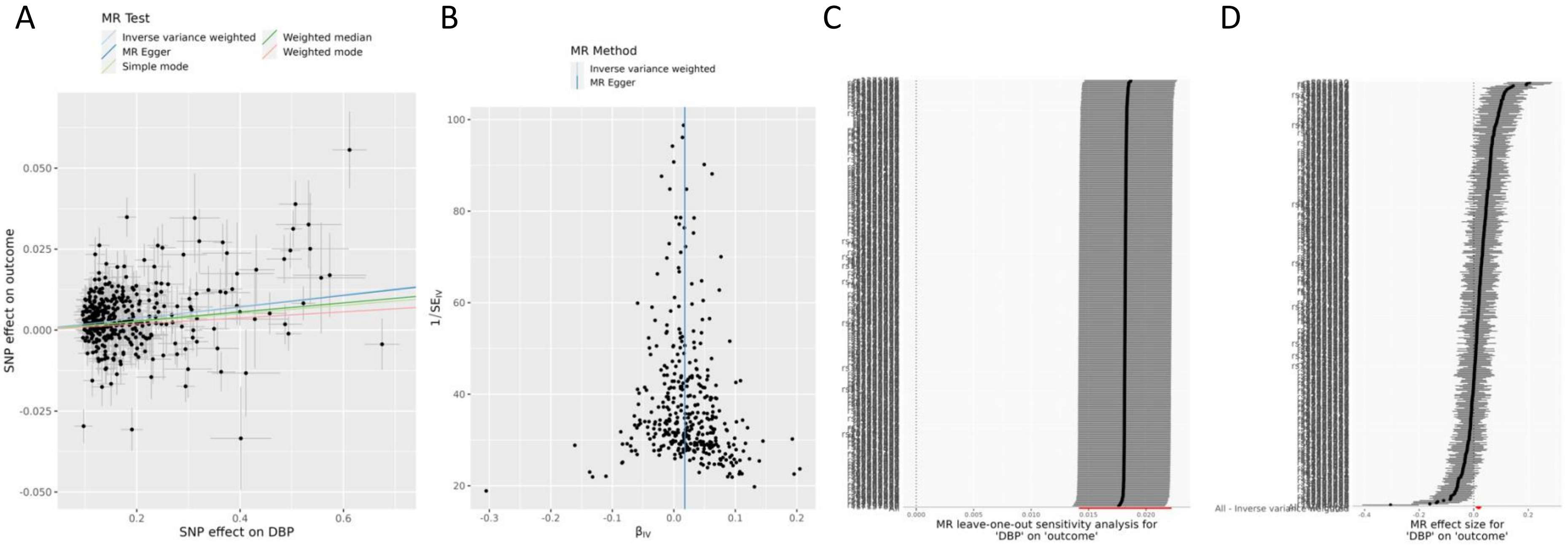

(A) A scatter plot showing SNP effects on the exposure and outcome. **(B)** A funnel plot of the association between exposure and outcome using each SNP individually against the inverse of the standard error of the causal effect. **(C)** Leave-one-out-analysis showing causal estimates after excluding each SNP from the analysis one-by-one. The IVW estimate using all SNPs is depicted in red. **(D)** Forest plot showing causal estimates using each SNP as a separate instrument (in black) and their combined effect using inverse-variance weighted and MR-Egger methods (in red).

Fig R. Causal effect of systolic blood pressure on multimorbidity.

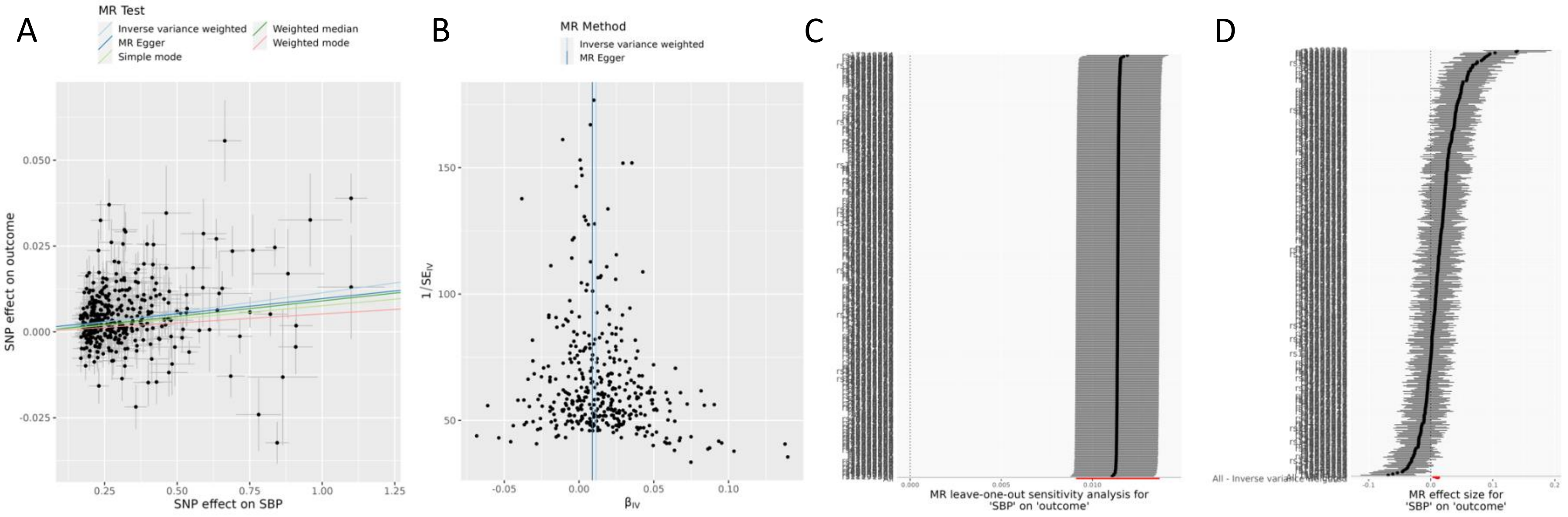

(A) A scatter plot showing SNP effects on the exposure and outcome. **(B)** A funnel plot of the association between exposure and outcome using each SNP individually against the inverse of the standard error of the causal effect. **(C)** Leave-one-out-analysis showing causal estimates after excluding each SNP from the analysis one-by-one. The IVW estimate using all SNPs is depicted in red. **(D)** Forest plot showing causal estimates using each SNP as a separate instrument (in black) and their combined effect using inverse-variance weighted and MR-Egger methods (in red).

Fig S. Causal effect of triglycerides on multimorbidity.

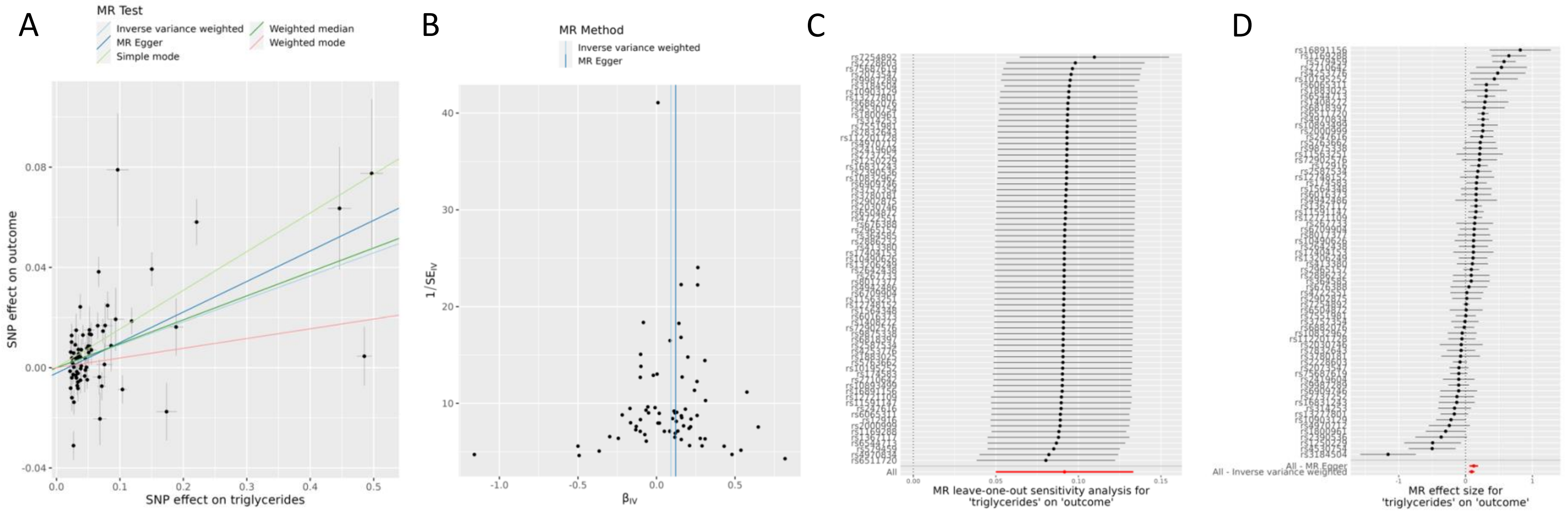

(A) A scatter plot showing SNP effects on the exposure and outcome. **(B)** A funnel plot of the association between exposure and outcome using each SNP individually against the inverse of the standard error of the causal effect. **(C)** Leave-one-out-analysis showing causal estimates after excluding each SNP from the analysis one-by-one. The IVW estimate using all SNPs is depicted in red. **(D)** Forest plot showing causal estimates using each SNP as a separate instrument (in black) and their combined effect using inverse-variance weighted and MR-Egger methods (in red).

Fig T. Causal effect of intelligence on multimorbidity.

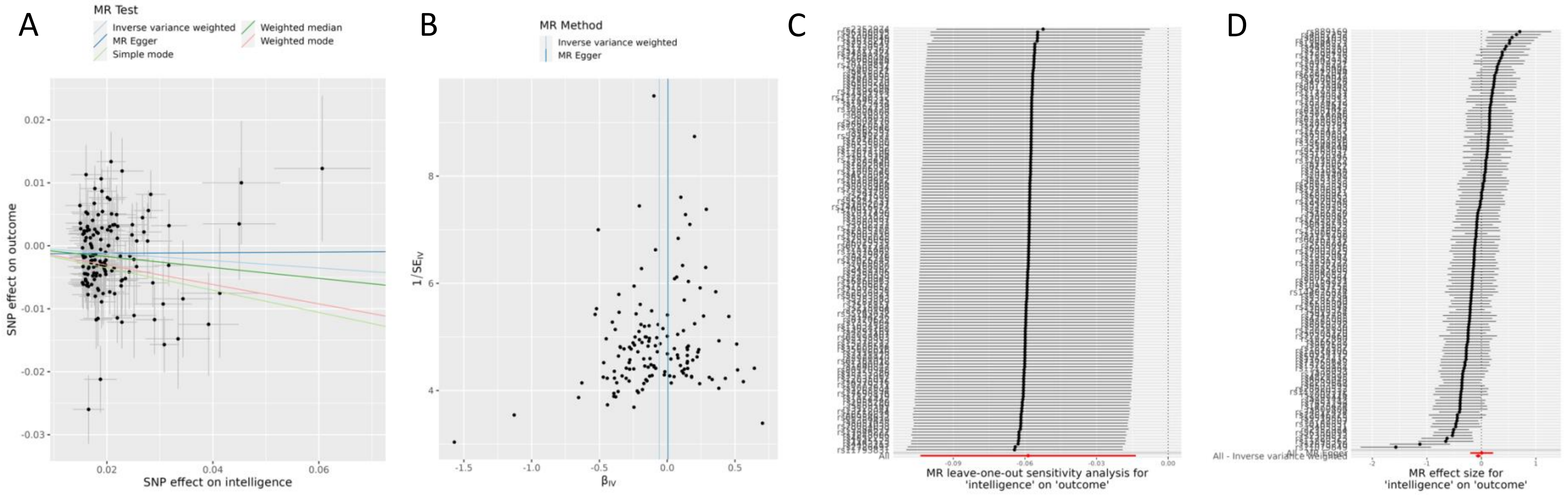

(A) A scatter plot showing SNP effects on the exposure and outcome. **(B)** A funnel plot of the association between exposure and outcome using each SNP individually against the inverse of the standard error of the causal effect. **(C)** Leave-one-out-analysis showing causal estimates after excluding each SNP from the analysis one-by-one. The IVW estimate using all SNPs is depicted in red. **(D)** Forest plot showing causal estimates using each SNP as a separate instrument (in black) and their combined effect using inverse-variance weighted and MR-Egger methods (in red).

Fig U. Causal effect of worry on multimorbidity.

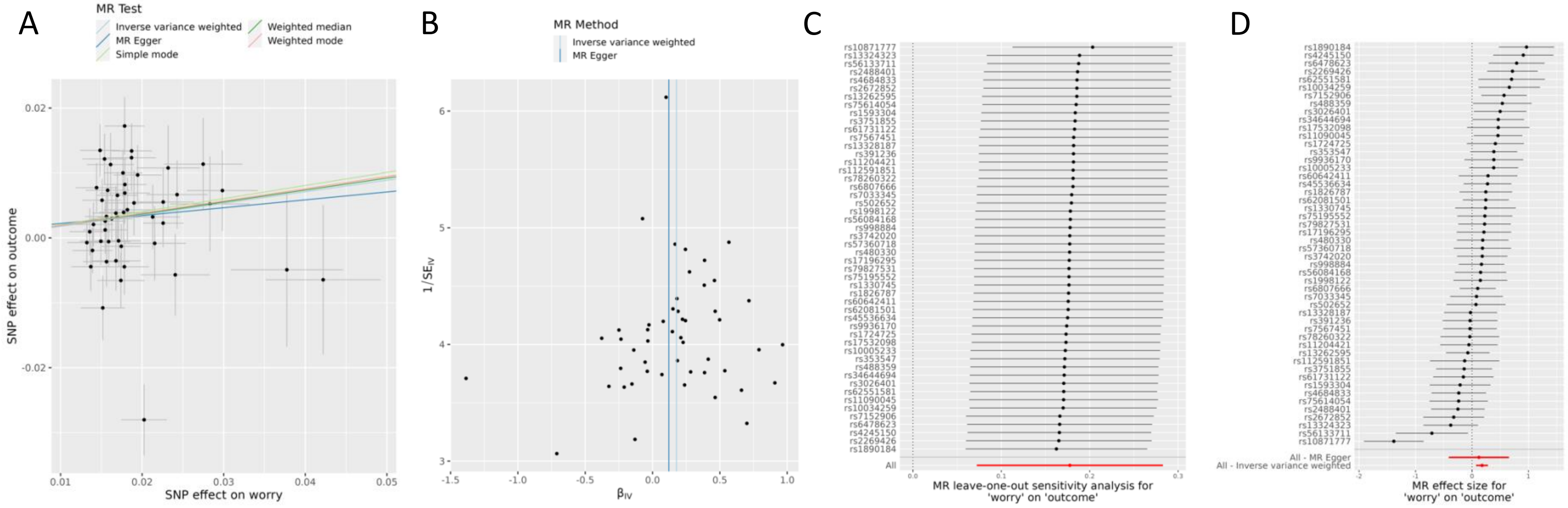

(A) A scatter plot showing SNP effects on the exposure and outcome. **(B)** A funnel plot of the association between exposure and outcome using each SNP individually against the inverse of the standard error of the causal effect. **(C)** Leave-one-out-analysis showing causal estimates after excluding each SNP from the analysis one-by-one. The IVW estimate using all SNPs is depicted in red. **(D)** Forest plot showing causal estimates using each SNP as a separate instrument (in black) and their combined effect using inverse-variance weighted and MR-Egger methods (in red).

Fig V. Causal effect of SESA on multimorbidity.

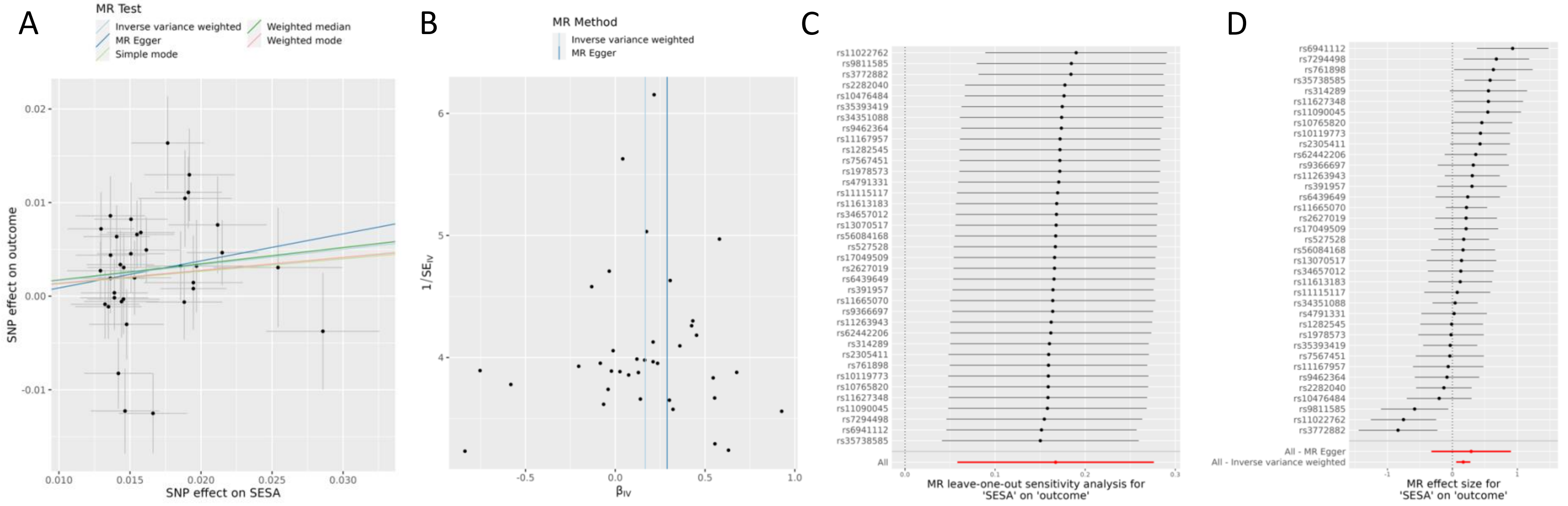

(A) A scatter plot showing SNP effects on the exposure and outcome. **(B)** A funnel plot of the association between exposure and outcome using each SNP individually against the inverse of the standard error of the causal effect. **(C)** Leave-one-out-analysis showing causal estimates after excluding each SNP from the analysis one-by-one. The IVW estimate using all SNPs is depicted in red. **(D)** Forest plot showing causal estimates using each SNP as a separate instrument (in black) and their combined effect using inverse-variance weighted and MR-Egger methods (in red). SESA; sensitivity to environmental stress and adversity.

Fig W. Causal effect of HDL cholesterol on multimorbidity.

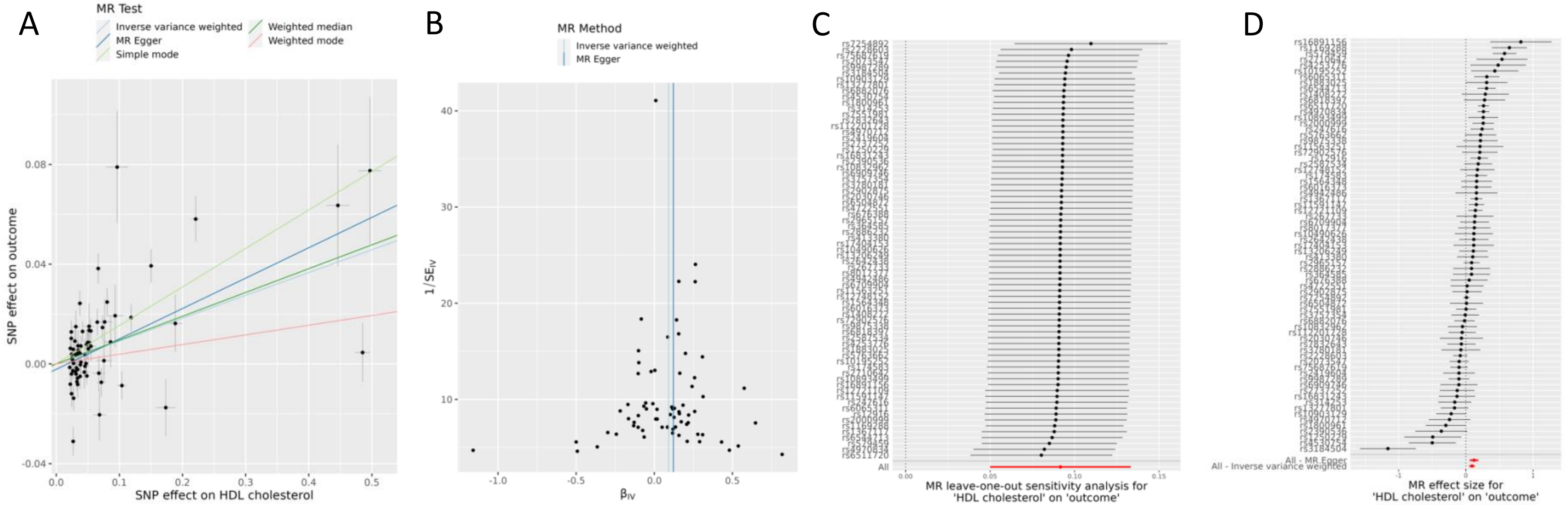

(A) A scatter plot showing SNP effects on the exposure and outcome. **(B)** A funnel plot of the association between exposure and outcome using each SNP individually against the inverse of the standard error of the causal effect. **(C)** Leave-one-out-analysis showing causal estimates after excluding each SNP from the analysis one-by-one. The IVW estimate using all SNPs is depicted in red. **(D)** Forest plot showing causal estimates using each SNP as a separate instrument (in black) and their combined effect using inverse-variance weighted and MR-Egger methods (in red). HDL, high-density lipoprotein.

Fig X. Causal effect of smoking status (current) on multimorbidity.

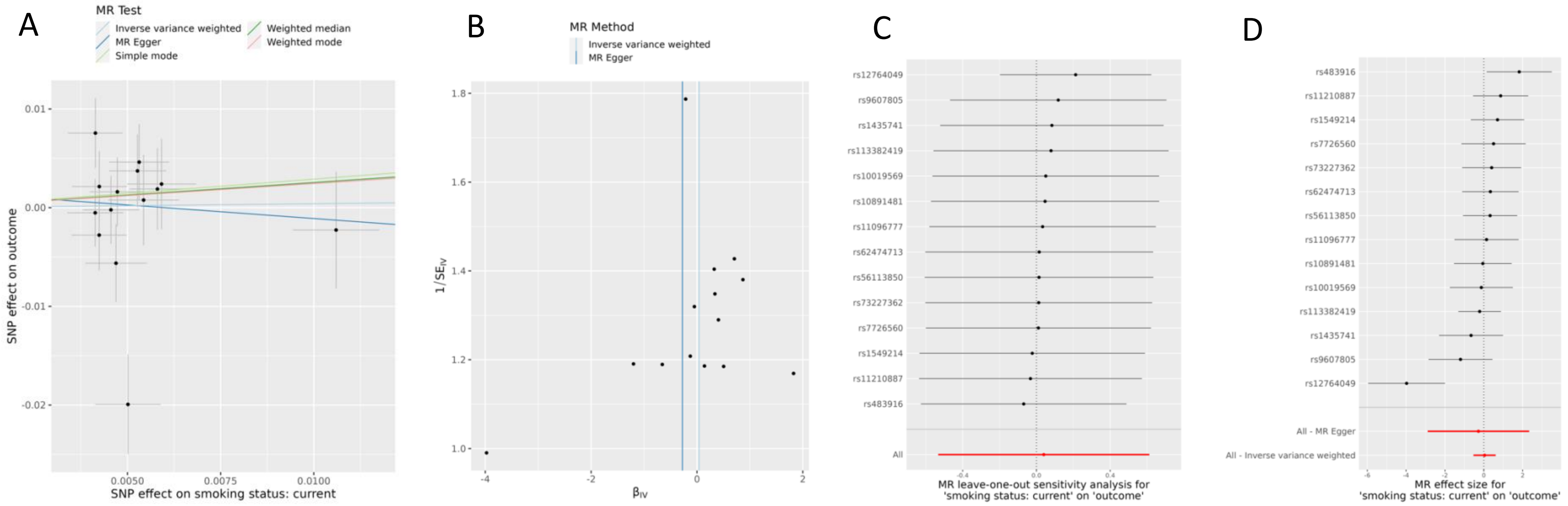

(A) A scatter plot showing SNP effects on the exposure and outcome. **(B)** A funnel plot of the association between exposure and outcome using each SNP individually against the inverse of the standard error of the causal effect. **(C)** Leave-one-out-analysis showing causal estimates after excluding each SNP from the analysis one-by-one. The IVW estimate using all SNPs is depicted in red. **(D)** Forest plot showing causal estimates using each SNP as a separate instrument (in black) and their combined effect using inverse-variance weighted and MR-Egger methods (in red).

Fig Y. Causal effect of c-reactive protein on multimorbidity.

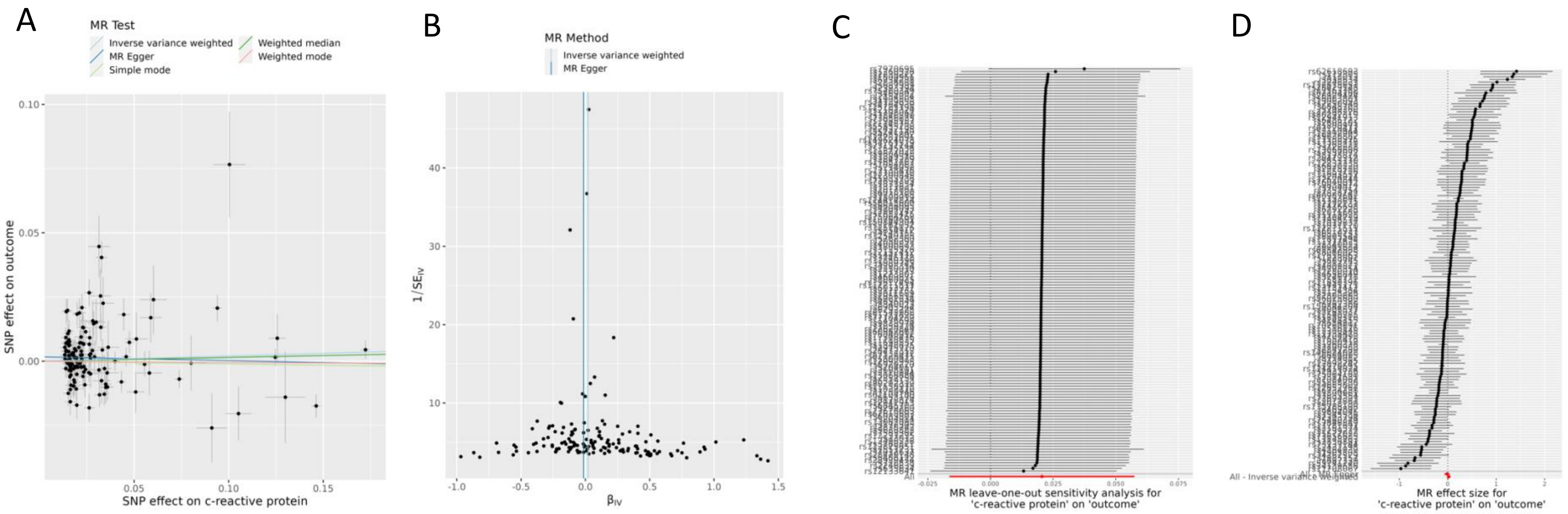

(A) A scatter plot showing SNP effects on the exposure and outcome. **(B)** A funnel plot of the association between exposure and outcome using each SNP individually against the inverse of the standard error of the causal effect. **(C)** Leave-one-out-analysis showing causal estimates after excluding each SNP from the analysis one-by-one. The IVW estimate using all SNPs is depicted in red. **(D)** Forest plot showing causal estimates using each SNP as a separate instrument (in black) and their combined effect using inverse-variance weighted and MR-Egger methods (in red).
