# Supplementary material for: A multivariate genome-wide association study of psycho-cardiometabolic multimorbidity
Source: PLoS Genet. 2023 Jun 30;19(6):e1010508. doi: 10.1371/journal.pgen.1010508 (PMC10343069; doi:10.1371/journal.pgen.1010508)
Supplement: S1 Text — (DOCX) [file pgen.1010508.s016.docx]

**S1 Text. FUMA SNP2GENE parameters**

[jobinfo]

created_at = 2023-03-18 16:36:22

title = mm_revision_0.1threshold_noGC_NikMahUKBB_FULL_562507_non-het

[version]

FUMA = v1.5.2

MAGMA = v1.08

GWAScatalog = e0_r2022-11-29

ANNOVAR = 2017-07-17

[inputfiles]

gwasfile = multimorbidity_NikMahUKBB_noGC_revision_FULL_keycolumns_non-het.txt.gz

chrcol = NA

poscol = NA

rsIDcol = SNP

pcol = P

eacol = A1

neacol = A2

orcol = NA

becol = BETA

secol = SE

leadSNPsfile = NA

addleadSNPs = 1

regionsfile = NA

[params]

N = 562507

Ncol = NA

exMHC = 1

MHCopt = annot

extMHC = NA

ensembl = v92

genetype = protein_coding

leadP = 5e-8

gwasP = 0.05

r2 = 0.1

r2_2 = 0.1

refpanel = 1KG/Phase3

pop = EUR

MAF = 0

refSNPs = 1

mergeDist = 250

[magma]

magma = 1

magma_window = 10

magma_exp = GTEx/v8/gtex_v8_ts_avg_log2TPM:GTEx/v8/gtex_v8_ts_general_avg_log2TPM

[posMap]

posMap = 1

posMapWindowSize = 10

posMapAnnot = NA

posMapCADDth = 0

posMapRDBth = NA

posMapChr15 = NA

posMapChr15Max = NA

posMapChr15Meth = NA

posMapAnnoDs = PsychENCODE/enhancer.bed.gz:PsychENCODE/enhancer_high_conf.bed.gz:PsychENCODE/PFC_H3K27ac_peak.bed.gz:PsychENCODE/TC_H3K27ac_peak.bed.gz:PsychENCODE/CBC_H3K27ac_peak.bed.gz:PsychENCODE/TARs.bed.gz:FANTOM5/TSS.bed.gz:FANTOM5/permissive_CAGE_peak.bed.gz:FANTOM5/robust_CAGE_peak.bed.gz:FANTOM5/permissive_enhancer.bed.gz:FANTOM5/robust_enhancer.bed.gz:BOCA/DLPFC_neuron.bed.gz:BOCA/DLPFC_glia.bed.gz:BOCA/OFC_neuron.bed.gz:BOCA/OFC_glia.bed.gz:BOCA/VLPFC_neuron.bed.gz:BOCA/VLPFC_glia.bed.gz:BOCA/ACC_neuron.bed.gz:BOCA/ACC_glia.bed.gz:BOCA/STC_neuron.bed.gz:BOCA/STC_glia.bed.gz:BOCA/ITC_neuron.bed.gz:BOCA/ITC_glia.bed.gz:BOCA/PMC_neuron.bed.gz:BOCA/PMC_glia.bed.gz:BOCA/INS_neuron.bed.gz:BOCA/INS_glia.bed.gz:BOCA/PVC_neuron.bed.gz:BOCA/PVC_glia.bed.gz:BOCA/AMY_neuron.bed.gz:BOCA/AMY_glia.bed.gz:BOCA/HIPP_neuron.bed.gz:BOCA/HIPP_glia.bed.gz:BOCA/MDT_neuron.bed.gz:BOCA/MDT_glia.bed.gz:BOCA/NAC_neuron.bed.gz:BOCA/NAC_glia.bed.gz:BOCA/PUT_neuron.bed.gz:BOCA/PUT_glia.bed.gz

posMapAnnoMeth = NA

[eqtlMap]

eqtlMap = 1

eqtlMaptss = eQTLcatalogue/Alasoo_2018_ge_macrophage_IFNg_Salmonella.txt.gz:eQTLcatalogue/Alasoo_2018_ge_macrophage_IFNg.txt.gz:eQTLcatalogue/Alasoo_2018_ge_macrophage_naive.txt.gz:eQTLcatalogue/Alasoo_2018_ge_macrophage_Salmonella.txt.gz:eQTLcatalogue/BLUEPRINT_ge_monocyte.txt.gz:eQTLcatalogue/BLUEPRINT_ge_neutrophil.txt.gz:eQTLcatalogue/BLUEPRINT_ge_T-cell.txt.gz:eQTLcatalogue/BrainSeq_ge_brain.txt.gz:eQTLcatalogue/CEDAR_B-cell_CD19.txt.gz:eQTLcatalogue/CEDAR_ileum.txt.gz:eQTLcatalogue/CEDAR_monocyte_CD14.txt.gz:eQTLcatalogue/CEDAR_neutrophil_CD15.txt.gz:eQTLcatalogue/CEDAR_platelet.txt.gz:eQTLcatalogue/CEDAR_rectum.txt.gz:eQTLcatalogue/CEDAR_T-cell_CD4.txt.gz:eQTLcatalogue/CEDAR_T-cell_CD8.txt.gz:eQTLcatalogue/CEDAR_transverse_colon.txt.gz:eQTLcatalogue/Fairfax_2012_B-cell_CD19.txt.gz:eQTLcatalogue/Fairfax_2014_IFN24.txt.gz:eQTLcatalogue/Fairfax_2014_LPS24.txt.gz:eQTLcatalogue/Fairfax_2014_LPS2.txt.gz:eQTLcatalogue/Fairfax_2014_naive.txt.gz:eQTLcatalogue/GENCORD_ge_fibroblast.txt.gz:eQTLcatalogue/GENCORD_ge_LCL.txt.gz:eQTLcatalogue/GENCORD_ge_T-cell.txt.gz:eQTLcatalogue/GEUVADIS_ge_LCL.txt.gz:eQTLcatalogue/HipSci_ge_iPSC.txt.gz:eQTLcatalogue/Kasela_2017_T-cell_CD4.txt.gz:eQTLcatalogue/Kasela_2017_T-cell_CD8.txt.gz:eQTLcatalogue/Lepik_2017_ge_blood.txt.gz:eQTLcatalogue/Naranbhai_2015_neutrophil_CD16.txt.gz:eQTLcatalogue/Nedelec_2016_ge_macrophage_Listeria.txt.gz:eQTLcatalogue/Nedelec_2016_ge_macrophage_naive.txt.gz:eQTLcatalogue/Nedelec_2016_ge_macrophage_Salmonella.txt.gz:eQTLcatalogue/Quach_2016_ge_monocyte_IAV.txt.gz:eQTLcatalogue/Quach_2016_ge_monocyte_LPS.txt.gz:eQTLcatalogue/Quach_2016_ge_monocyte_naive.txt.gz:eQTLcatalogue/Quach_2016_ge_monocyte_Pam3CSK4.txt.gz:eQTLcatalogue/Quach_2016_ge_monocyte_R848.txt.gz:eQTLcatalogue/Schwartzentruber_2018_ge_sensory_neuron.txt.gz:eQTLcatalogue/TwinsUK_ge_blood.txt.gz:eQTLcatalogue/TwinsUK_ge_fat.txt.gz:eQTLcatalogue/TwinsUK_ge_LCL.txt.gz:eQTLcatalogue/TwinsUK_ge_skin.txt.gz:eQTLcatalogue/van_de_Bunt_2015_ge_pancreatic_islet.txt.gz:PsychENCODE/PsychENCODE_eQTLs.txt.gz:scRNA_eQTLs/B_cell.txt.gz:scRNA_eQTLs/DC.txt.gz:scRNA_eQTLs/NK.txt.gz:scRNA_eQTLs/Monocyte.txt.gz:scRNA_eQTLs/Classical_Monocyte.txt.gz:scRNA_eQTLs/Non_classical_Monocyte.txt.gz:scRNA_eQTLs/T_CD4.txt.gz:scRNA_eQTLs/T_CD8.txt.gz:scRNA_eQTLs/PBMC.txt.gz:DICE/B_cell_naive.txt.gz:DICE/T_CD4_naive.txt.gz:DICE/T_CD4_naive_activated.txt.gz:DICE/T_CD8_naive.txt.gz:DICE/T_CD8_naive_activated.txt.gz:DICE/Monocyte_classical.txt.gz:DICE/Monocyte_non_classical.txt.gz:DICE/NK.txt.gz:DICE/T_CD4_TFH.txt.gz:DICE/T_CD4_TH1.txt.gz:DICE/T_CD4_TH17.txt.gz:DICE/T_CD4_TH1_17.txt.gz:DICE/T_CD4_TH2.txt.gz:DICE/T_CD4_memory_TREG.txt.gz:DICE/T_CD4_naive_TREG.txt.gz:eQTLGen/eQTLGen_cis_eQTLs.txt.gz:eQTLGen/eQTLGen_trans_eQTLs.txt.gz:BloodeQTL/BloodeQTL.txt.gz:BIOSQTL/BIOS_eQTL_geneLevel.txt.gz:MuTHER/MuTHER_Adipose.txt.gz:MuTHER/MuTHER_LCL.txt.gz:MuTHER/MuTHER_Skin.txt.gz:xQTLServer/xQTLServer_eQTLs.txt.gz:CMC/CMC_SVA_cis.txt.gz:CMC/CMC_SVA_trans.txt.gz:CMC/CMC_NoSVA_cis.txt.gz:CMC/CMC_NoSVA_trans.txt.gz:BRAINEAC/CRBL.txt.gz:BRAINEAC/FCTX.txt.gz:BRAINEAC/HIPP.txt.gz:BRAINEAC/MEDU.txt.gz:BRAINEAC/OCTX.txt.gz:BRAINEAC/PUTM.txt.gz:BRAINEAC/SNIG.txt.gz:BRAINEAC/TCTX.txt.gz:BRAINEAC/THAL.txt.gz:BRAINEAC/WHMT.txt.gz:BRAINEAC/aveALL.txt.gz:GTEx/v8/Adipose_Subcutaneous.txt.gz:GTEx/v8/Adipose_Visceral_Omentum.txt.gz:GTEx/v8/Adrenal_Gland.txt.gz:GTEx/v8/Cells_EBV-transformed_lymphocytes.txt.gz:GTEx/v8/Whole_Blood.txt.gz:GTEx/v8/Artery_Aorta.txt.gz:GTEx/v8/Artery_Coronary.txt.gz:GTEx/v8/Artery_Tibial.txt.gz:GTEx/v8/Brain_Amygdala.txt.gz:GTEx/v8/Brain_Anterior_cingulate_cortex_BA24.txt.gz:GTEx/v8/Brain_Caudate_basal_ganglia.txt.gz:GTEx/v8/Brain_Cerebellar_Hemisphere.txt.gz:GTEx/v8/Brain_Cerebellum.txt.gz:GTEx/v8/Brain_Cortex.txt.gz:GTEx/v8/Brain_Frontal_Cortex_BA9.txt.gz:GTEx/v8/Brain_Hippocampus.txt.gz:GTEx/v8/Brain_Hypothalamus.txt.gz:GTEx/v8/Brain_Nucleus_accumbens_basal_ganglia.txt.gz:GTEx/v8/Brain_Putamen_basal_ganglia.txt.gz:GTEx/v8/Brain_Spinal_cord_cervical_c-1.txt.gz:GTEx/v8/Brain_Substantia_nigra.txt.gz:GTEx/v8/Breast_Mammary_Tissue.txt.gz:GTEx/v8/Colon_Sigmoid.txt.gz:GTEx/v8/Colon_Transverse.txt.gz:GTEx/v8/Esophagus_Gastroesophageal_Junction.txt.gz:GTEx/v8/Esophagus_Mucosa.txt.gz:GTEx/v8/Esophagus_Muscularis.txt.gz:GTEx/v8/Heart_Atrial_Appendage.txt.gz:GTEx/v8/Heart_Left_Ventricle.txt.gz:GTEx/v8/Kidney_Cortex.txt.gz:GTEx/v8/Liver.txt.gz:GTEx/v8/Lung.txt.gz:GTEx/v8/Muscle_Skeletal.txt.gz:GTEx/v8/Nerve_Tibial.txt.gz:GTEx/v8/Ovary.txt.gz:GTEx/v8/Pancreas.txt.gz:GTEx/v8/Pituitary.txt.gz:GTEx/v8/Prostate.txt.gz:GTEx/v8/Minor_Salivary_Gland.txt.gz:GTEx/v8/Cells_Cultured_fibroblasts.txt.gz:GTEx/v8/Skin_Not_Sun_Exposed_Suprapubic.txt.gz:GTEx/v8/Skin_Sun_Exposed_Lower_leg.txt.gz:GTEx/v8/Small_Intestine_Terminal_Ileum.txt.gz:GTEx/v8/Spleen.txt.gz:GTEx/v8/Stomach.txt.gz:GTEx/v8/Testis.txt.gz:GTEx/v8/Thyroid.txt.gz:GTEx/v8/Uterus.txt.gz:GTEx/v8/Vagina.txt.gz:GTEx/v7/Adipose_Subcutaneous.txt.gz:GTEx/v7/Adipose_Visceral_Omentum.txt.gz:GTEx/v7/Adrenal_Gland.txt.gz:GTEx/v7/Cells_EBV-transformed_lymphocytes.txt.gz:GTEx/v7/Whole_Blood.txt.gz:GTEx/v7/Artery_Aorta.txt.gz:GTEx/v7/Artery_Coronary.txt.gz:GTEx/v7/Artery_Tibial.txt.gz:GTEx/v7/Brain_Amygdala.txt.gz:GTEx/v7/Brain_Anterior_cingulate_cortex_BA24.txt.gz:GTEx/v7/Brain_Caudate_basal_ganglia.txt.gz:GTEx/v7/Brain_Cerebellar_Hemisphere.txt.gz:GTEx/v7/Brain_Cerebellum.txt.gz:GTEx/v7/Brain_Cortex.txt.gz:GTEx/v7/Brain_Frontal_Cortex_BA9.txt.gz:GTEx/v7/Brain_Hippocampus.txt.gz:GTEx/v7/Brain_Hypothalamus.txt.gz:GTEx/v7/Brain_Nucleus_accumbens_basal_ganglia.txt.gz:GTEx/v7/Brain_Putamen_basal_ganglia.txt.gz:GTEx/v7/Brain_Spinal_cord_cervical_c-1.txt.gz:GTEx/v7/Brain_Substantia_nigra.txt.gz:GTEx/v7/Breast_Mammary_Tissue.txt.gz:GTEx/v7/Colon_Sigmoid.txt.gz:GTEx/v7/Colon_Transverse.txt.gz:GTEx/v7/Esophagus_Gastroesophageal_Junction.txt.gz:GTEx/v7/Esophagus_Mucosa.txt.gz:GTEx/v7/Esophagus_Muscularis.txt.gz:GTEx/v7/Heart_Atrial_Appendage.txt.gz:GTEx/v7/Heart_Left_Ventricle.txt.gz:GTEx/v7/Liver.txt.gz:GTEx/v7/Lung.txt.gz:GTEx/v7/Muscle_Skeletal.txt.gz:GTEx/v7/Nerve_Tibial.txt.gz:GTEx/v7/Ovary.txt.gz:GTEx/v7/Pancreas.txt.gz:GTEx/v7/Pituitary.txt.gz:GTEx/v7/Prostate.txt.gz:GTEx/v7/Minor_Salivary_Gland.txt.gz:GTEx/v7/Cells_Transformed_fibroblasts.txt.gz:GTEx/v7/Skin_Not_Sun_Exposed_Suprapubic.txt.gz:GTEx/v7/Skin_Sun_Exposed_Lower_leg.txt.gz:GTEx/v7/Small_Intestine_Terminal_Ileum.txt.gz:GTEx/v7/Spleen.txt.gz:GTEx/v7/Stomach.txt.gz:GTEx/v7/Testis.txt.gz:GTEx/v7/Thyroid.txt.gz:GTEx/v7/Uterus.txt.gz:GTEx/v7/Vagina.txt.gz:GTEx/v6/Adipose_Subcutaneous.txt.gz:GTEx/v6/Adipose_Visceral_Omentum.txt.gz:GTEx/v6/Adrenal_Gland.txt.gz:GTEx/v6/Cells_EBV-transformed_lymphocytes.txt.gz:GTEx/v6/Whole_Blood.txt.gz:GTEx/v6/Artery_Aorta.txt.gz:GTEx/v6/Artery_Coronary.txt.gz:GTEx/v6/Artery_Tibial.txt.gz:GTEx/v6/Brain_Anterior_cingulate_cortex_BA24.txt.gz:GTEx/v6/Brain_Caudate_basal_ganglia.txt.gz:GTEx/v6/Brain_Cerebellar_Hemisphere.txt.gz:GTEx/v6/Brain_Cerebellum.txt.gz:GTEx/v6/Brain_Cortex.txt.gz:GTEx/v6/Brain_Frontal_Cortex_BA9.txt.gz:GTEx/v6/Brain_Hippocampus.txt.gz:GTEx/v6/Brain_Hypothalamus.txt.gz:GTEx/v6/Brain_Nucleus_accumbens_basal_ganglia.txt.gz:GTEx/v6/Brain_Putamen_basal_ganglia.txt.gz:GTEx/v6/Breast_Mammary_Tissue.txt.gz:GTEx/v6/Colon_Sigmoid.txt.gz:GTEx/v6/Colon_Transverse.txt.gz:GTEx/v6/Esophagus_Gastroesophageal_Junction.txt.gz:GTEx/v6/Esophagus_Mucosa.txt.gz:GTEx/v6/Esophagus_Muscularis.txt.gz:GTEx/v6/Heart_Atrial_Appendage.txt.gz:GTEx/v6/Heart_Left_Ventricle.txt.gz:GTEx/v6/Liver.txt.gz:GTEx/v6/Lung.txt.gz:GTEx/v6/Muscle_Skeletal.txt.gz:GTEx/v6/Nerve_Tibial.txt.gz:GTEx/v6/Ovary.txt.gz:GTEx/v6/Pancreas.txt.gz:GTEx/v6/Pituitary.txt.gz:GTEx/v6/Prostate.txt.gz:GTEx/v6/Cells_Transformed_fibroblasts.txt.gz:GTEx/v6/Skin_Not_Sun_Exposed_Suprapubic.txt.gz:GTEx/v6/Skin_Sun_Exposed_Lower_leg.txt.gz:GTEx/v6/Small_Intestine_Terminal_Ileum.txt.gz:GTEx/v6/Spleen.txt.gz:GTEx/v6/Stomach.txt.gz:GTEx/v6/Testis.txt.gz:GTEx/v6/Thyroid.txt.gz:GTEx/v6/Uterus.txt.gz:GTEx/v6/Vagina.txt.gz

eqtlMapSig = 1

eqtlMapP = 1

eqtlMapCADDth = 0

eqtlMapRDBth = NA

eqtlMapChr15 = NA

eqtlMapChr15Max = NA

eqtlMapChr15Meth = NA

eqtlMapAnnoDs = NA

eqtlMapAnnoMeth = NA

[ciMap]

ciMap = 1

ciMapBuiltin = EP/PsychENCODE/EP_links_oneway.txt.gz:HiC/PsychENCODE/Promoter_anchored_loops.txt.gz:EP/FANTOM5/EP_correlation_cell_type_oneway.txt.gz:EP/FANTOM5/EP_correlation_organ_oneway.txt.gz:HiC/Giusti-Rodriguez_et_al_2019/Adult_Cortex.txt.gz:HiC/Giusti-Rodriguez_et_al_2019/Fetal_Cortex.txt.gz:HiC/GSE87112/Adrenal.txt.gz:HiC/GSE87112/Aorta.txt.gz:HiC/GSE87112/Bladder.txt.gz:HiC/GSE87112/Dorsolateral_Prefrontal_Cortex.txt.gz:HiC/GSE87112/Hippocampus.txt.gz:HiC/GSE87112/Left_Ventricle.txt.gz:HiC/GSE87112/Liver.txt.gz:HiC/GSE87112/Lung.txt.gz:HiC/GSE87112/Ovary.txt.gz:HiC/GSE87112/Pancreas.txt.gz:HiC/GSE87112/Psoas.txt.gz:HiC/GSE87112/Right_Ventricle.txt.gz:HiC/GSE87112/Small_Bowel.txt.gz:HiC/GSE87112/Spleen.txt.gz:HiC/GSE87112/GM12878.txt.gz:HiC/GSE87112/IMR90.txt.gz:HiC/GSE87112/Mesenchymal_Stem_Cell.txt.gz:HiC/GSE87112/Mesendoderm.txt.gz:HiC/GSE87112/Neural_Progenitor_Cell.txt.gz:HiC/GSE87112/Trophoblast-like_Cell.txt.gz:HiC/GSE87112/hESC.txt.gz

ciMapFileN = 0

ciMapFiles = NA

ciMapFDR = 1e-6

ciMapPromWindow = 250-500

ciMapRoadmap = NA

ciMapEnhFilt = 0

ciMapPromFilt = 0

ciMapCADDth = 0

ciMapRDBth = NA

ciMapChr15 = NA

ciMapChr15Max = NA

ciMapChr15Meth = NA

ciMapAnnoDs = NA

ciMapAnnoMeth = NA
