# Supplementary material for: A multivariate genome-wide association study of psycho-cardiometabolic multimorbidity
Source: PLoS Genet. 2023 Jun 30;19(6):e1010508. doi: 10.1371/journal.pgen.1010508 (PMC10343069; doi:10.1371/journal.pgen.1010508)
Supplement: S2 Text — (DOCX) [file pgen.1010508.s017.docx]

**S2 Text. FUMA GENE2FUNC parameters**

[jobinfo]

created_at=2023-03-18 17:08:26

title=None

snp2geneID=239737

snp2geneTitle=mm_revision_0.1threshold_noGC_NikMahUKBB_FULL_562507_non-het

[params]

gtype=text

gval=ENSG00000142657:ENSG00000116761:ENSG00000050628:ENSG00000132485:ENSG00000172260:ENSG00000162620:ENSG00000254685:ENSG00000259030:ENSG00000116783:ENSG00000162621:ENSG00000116791:ENSG00000162623:ENSG00000162624:ENSG00000117054:ENSG00000117174:ENSG00000184611:ENSG00000168237:ENSG00000129071:ENSG00000181788:ENSG00000113391:ENSG00000113758:ENSG00000111846:ENSG00000111837:ENSG00000124827:ENSG00000197977:ENSG00000224531:ENSG00000244476:ENSG00000205269:ENSG00000111863:ENSG00000095951:ENSG00000078401:ENSG00000112137:ENSG00000145979:ENSG00000145990:ENSG00000187461:ENSG00000124523:ENSG00000225921:ENSG00000164694:ENSG00000112096:ENSG00000146457:ENSG00000120437:ENSG00000120438:ENSG00000112110:ENSG00000146453:ENSG00000130368:ENSG00000197081:ENSG00000175003:ENSG00000112499:ENSG00000146477:ENSG00000198670:ENSG00000122194:ENSG00000085511:ENSG00000026652:ENSG00000185345:ENSG00000112530:ENSG00000225683:ENSG00000112531:ENSG00000106443:ENSG00000005108:ENSG00000106460:ENSG00000146530:ENSG00000006747:ENSG00000122644:ENSG00000006468:ENSG00000136193:ENSG00000136573:ENSG00000164808:ENSG00000147872:ENSG00000177047:ENSG00000137080:ENSG00000236637:ENSG00000214042:ENSG00000186803:ENSG00000147885:ENSG00000234829:ENSG00000228083:ENSG00000147873:ENSG00000198642:ENSG00000120235:ENSG00000197919:ENSG00000184995:ENSG00000099810:ENSG00000264545:ENSG00000224854:ENSG00000147889:ENSG00000147883:ENSG00000107105:ENSG00000125485:ENSG00000125484:ENSG00000148308:ENSG00000170835:ENSG00000160271:ENSG00000148288:ENSG00000171102:ENSG00000148296:ENSG00000148297:ENSG00000148303:ENSG00000148290:ENSG00000148291:ENSG00000148248:ENSG00000198870:ENSG00000148300:ENSG00000160323:ENSG00000160325:ENSG00000160326:ENSG00000187616:ENSG00000197859:ENSG00000196990:ENSG00000123454:ENSG00000123453:ENSG00000160293:ENSG00000169925:ENSG00000107736:ENSG00000122966:ENSG00000135127:ENSG00000089154:ENSG00000089159:ENSG00000089163:ENSG00000170890:ENSG00000135097:ENSG00000111775:ENSG00000111780:ENSG00000170855:ENSG00000257218:ENSG00000111786:ENSG00000088986:ENSG00000110871:ENSG00000022840:ENSG00000167272:ENSG00000157782:ENSG00000110917:ENSG00000175970:ENSG00000122971:ENSG00000157837:ENSG00000272214:ENSG00000135100:ENSG00000157895:ENSG00000135114:ENSG00000089041:ENSG00000135124:ENSG00000110931:ENSG00000089094:ENSG00000182500:ENSG00000139714:ENSG00000139725:ENSG00000139718:ENSG00000110801:ENSG00000158023:ENSG00000100448:ENSG00000166068:ENSG00000086506:ENSG00000131634:ENSG00000108424:ENSG00000198933:ENSG00000159111:ENSG00000141295:ENSG00000189120:ENSG00000167182:ENSG00000108439:ENSG00000167183:ENSG00000108465:ENSG00000005243:ENSG00000082641:ENSG00000108468:ENSG00000002919:ENSG00000141293:ENSG00000120094:ENSG00000173917:ENSG00000120093:ENSG00000182742:ENSG00000120075:ENSG00000108511:ENSG00000260027:ENSG00000120068:ENSG00000170689:ENSG00000159182:ENSG00000159184:ENSG00000170703:ENSG00000136436:ENSG00000159199:ENSG00000159202:ENSG00000159210:ENSG00000159224:ENSG00000159217:ENSG00000167080:ENSG00000167083:ENSG00000108798:ENSG00000173868:ENSG00000198740:ENSG00000262039:ENSG00000167085:ENSG00000064300:ENSG00000182575:ENSG00000121067:ENSG00000005882:ENSG00000134755:ENSG00000196628:ENSG00000060069:ENSG00000101425:ENSG00000100985

bkgtype=select

bkgval=protein_coding

MHC=1

ensembl=v92

gsFileN=0

gsFiles=NA

gene_exp=GTEx/v8/gtex_v8_ts_avg_log2TPM:GTEx/v8/gtex_v8_ts_general_avg_log2TPM

adjPmeth=fdr_bh

adjPcut=0.05

minOverlap=2
