# Supplementary material for: A multivariate genome-wide association study of psycho-cardiometabolic multimorbidity
Source: PLoS Genet. 2023 Jun 30;19(6):e1010508. doi: 10.1371/journal.pgen.1010508 (PMC10343069; doi:10.1371/journal.pgen.1010508)
Supplement: Attachment — Submitted filename: Reviewer-response-multimorbidity-GWAS.docx [file pgen.1010508.s018.docx]

April 14, 2023

Dear Prof Barsh and Prof Copenhaver,

Please find enclosed our revised manuscript, entitled “A multivariate genome-wide association study of psycho-cardiometabolic multimorbidity”, for consideration for publication in PLOS Genetics.

As recommended by the reviewers, we:

- reran the multivariate GWAS and all follow-up analyses using the newer and larger CAD GWAS to improve power and statistical precision;
- added sex-specific polygenic risk score analyses;
- conducted genetic correlation and Mendelian randomization analyses to examine associations between selected risk factors and multimorbidity.

Our findings indicate that multimorbidity between coronary artery disease, type 2 diabetes and depression may be partly accounted for by shared genetic aetiology, with the potential involvement of immunoinflammatory pathways.

We believe that this manuscript has substantially improved during the review process and would like to thank the reviewers for their valuable feedback.

Please note that we would like to make this publication open access (gold route). The University of Bath has an Open Access agreement with Plos Genetics in place. For the purpose of open access, the author has applied a Creative Commons Attribution (CC-BY) licence to any Author Accepted Manuscript version arising.

Thank you in advance for your consideration of this revision.

Sincerely,

Esther Walton,

on behalf of all the authors

**Response to Reviewers’ Comments**

We thank the Reviewers for their comments and suggestions. The Reviewers’ comments are in **bold**. Our responses are in regular font (in blue). In *italics* we include the updated excerpts from the manuscript.

**Reviewer #1**

**R1.1 Important methodological advance: This paper is an interesting advance to the rapidly expanding field of “multimorbidity” – usually defined as the study of patients with more than one long term condition. The paper uses a genomic SEM approach to define a “latent phenotype” that is based on the overlap between three common conditions – type 2 diabetes, heart disease and depression. They then perform a GWAS of this latent phenotype and identify associated variants. Critically , the authors take the analysis a step further and use a heterogeneity statistic to assess whether or not variants associated with their latent multimorbidity phenotype are likely to be genuinely contributing to it independently of main effects on one of the traits. This is an important methodological advance for the field, but the approach looks like it has not resulted in a large number of new, genuine “multi-morbidity” signals. The well known signals on chromosome 9 that are involved in diabetes and heart disease look convincing, but most of the variants show some sign that they are mainly driven by one of the 3 traits – according to the heterogeneity stats in the supplementary tables. The data in table 3 is more promising – a multi morbidity polygenic risk score is increasing the risk of having all 3 conditions more than having just two of them. This result suggests the variants are capturing some of the depression component not just the CAD and T2D components to the latent phenotype.**

We thank Reviewer 1 for their encouraging comment highlighting our methodological approach and the polygenic risk score results in particular.

**R1.2 CAD GWAS: The CAD data used needs updating. The Nikpay et al. paper is I believe the largest GWAS of CAD without including UK Biobank, and includes 60,000 cases: PMID: 26343387. This will increase power substantially from the Schunkert et al. data using 22,000 cases – especially as the statistical confidence of some of the genetic correlations looks relatively weak.**

We thank Reviewer 1 (and 3) for highlighting this important issue. We agree that including the newer and larger CAD GWAS (Nikpay et al., 2015) instead of (Schunkert et al., 2011) would increase power substantially.

Initially, we decided against this newer GWAS, as it might introduce concerns regarding ancestry. As stated by the developers of Genomic SEM on their GitHub wiki: “In order for ld-score regression to produce accurate results **it is critical that the user both include only summary statistics that were calculated within a single ethnic group** AND that these summary statistics are matched with LD scores for the same ethnicity” (see <https://github.com/GenomicSEM/GenomicSEM/wiki/2.-Important-resources-and-key-information>). Given that Nikpay et al. is based on individuals from mixed ancestry groups (with 77% being European), we initially decided to reduce potential ancestry bias and use a more homogeneous, but less powered GWAS instead.

However, following recommendations by both Reviewer 1 and 3, we decided to rerun the analyses in the following two ways (see also Table R1):

1) include the largest GWAS for each trait for maximum power (GWAS_with.UKBB_)
   a) CAD: Nikpay et al., 2015
   b) T2D: Mahajan et al., 2018
   c) MD: Howard et al., 2019
2) include the largest GWAS for each trait excluding UK Biobank data (GWAS_without.UKBB_), which is used as a target set for polygenic risk score analyses
   a) CAD: Nikpay et al., 2015
   b) T2D: Scott et al., 2017
   c) MD: Howard et al., 2019 (excluding UK Biobank data)

| **Phenotype** | **Used in** | **GWAS** | **Year** | **Cases** | **Controls** | **includes UKBB** |
| --- | --- | --- | --- | --- | --- | --- |
| **CAD** | Discovery GWAS for Genomic SEM | Nikpay et al. | 2015 | 60,801 | 123,504 | no |
|  | Discovery GWAS for PRS | Nikpay et al. | 2015 | 60,801 | 123,504 | no |
| **T2D** | Discovery GWAS for GenomicSEM | Mahajan et al. | 2018 | 74,124 | 824,006 | yes |
|  | Discovery GWAS for PRS | Scott et al. | 2017 | 26,676 | 132,532 | no |
| **MD** | Discovery GWAS for GenomicSEM | Howard et al. | 2019 | 246,363 | 561,190 | yes |
|  | Discovery GWAS for PRS | Howard et al.  (no UKBB) | 2019 | 140,045 | 378,325 | no |

**Table R1. A list of Contributing Genome-Wide Association Studies.** Univariate GWASs contributing to the multivariate GWAS of psycho-cardiometabolic multimorbidity. All summary statistics are based on individuals of European ancestry, apart from Nikpay et al., which also includes individuals from mixed ancestry groups (with 77% being European). Summary statistics used to construct the PRS exclude the UK Biobank cohort. GWAS, genome-wide association study; PRS, polygenic risk score.

The genetic correlations between our original multimorbidity GWAS results (using Schunkert et al., 2011), GWAS_with.UKBB_, and GWAS_without.UKBB_ were very high, suggesting that all versions were capturing a similar genetic signal:

- GWAS_with.UKBB_ and original multimorbidity GWAS: *r*_g_ = 0.91 (*SE* = 0.01), *P* < 0.001
- GWAS_without.UKBB_ and original multimorbidity GWAS: *r*_g_ = 0.96 (*SE* = 0.01), *P* < 0.001
- GWAS_with.UKBB_ and GWAS_without.UKBB_: *r*_g_ = 0.98 (*SE* = 0.01), *P* < 0.001

To capitalize on power as suggested by Reviewer 1 and 3, we decided to prioritize GWAS_with.UKBB_ in the manuscript and use GWAS_without.UKBB_ for the follow-up PRS analyses. We were able to reproduce our initial findings:

Genetic Correlations

- **Original results:** in our original results, CAD was weakly-to-moderately correlated with depression (*r_g_* = 0.20, *SE* = 0.08, *P* = .019) and T2D (*r_g_* = 0.39, *SE* = 0.13, *P* = .003), whereas evidence for a genetic overlap between depression and T2D was weaker (*r_g_* = 0.13, *SE* = 0.07, *P* = .081).
- **Current results:** in the revised version, we observe comparable correlation magnitudes but smaller standard errors, giving us greater statistical confidence in our findings (*r_g_* _CAD-MD_ = 0.13, *SE* = 0.03, *P* = 3e-6; *r_g_* _CAD-T2D_ = 0.39, *SE* = 0.03, P = 2e-34; *r_g_* _T2D-MD_ = 0.15, *SE* = 0.02, *P* = 4e-15). Note that the correlation between T2D and depression is now significant.

Factor Analysis

Factor loadings were similar across the two versions (Figure R1). Although in the revised version, the strongest loading is observed for T2D rather than CAD.

**Figure R1. Common Factor Model for Psycho-Cardiometabolic Multimorbidity**. Standardized coefficients (*SE*) for the genetically defined common factor of multimorbidity. The model uses unit variance identification for the latent factor. MM, multimorbidity; CAD, coronary artery disease; T2D, type 2 diabetes; MD, major depression; U, residual variance.

Multivariate GWAS

- **Original results:** in our original GWAS 13 independent SNPs were identified at *r*^2^ < 0.6 and nine at the newly applied threshold of *r*^2^ < 0.1 (see R1.3).
- **Current results:** in the revised version, we identify 11 independent SNPs at *r*^2^ < 0.1. Genomic loci on chromosome 6 and 9 overlap with those identified in the original GWAS.

Gene Mapping

- **Original results:** a total of 71 unique genes were implicated, four of which were identified via all four distinct techniques: positional mapping, eQTL associations, chromatin interaction mapping and MAGMA gene-based analysis.
- **Current results:** in the revised version, a total of 200 unique genes are prioritised (as expected due to the enhanced power), 18 of which are implicated by all four techniques.

Polygenic risk score (PRS) analysis

- PRS results based on GWAS_without.UKBB_ align closely with original PRS results, with the new PRS being associated even stronger with phenotypic multimorbidity (OR = 1.91 vs. OR = 1.70).

We feel that these updated results confirm our original findings and improve precision. In the manuscript, we updated the methods (pages 6-11, and Table 1) and results section (pages 11-20, Figure 1-3, and Table 2-4) accordingly. Considering that these updated results are now based on much larger, but slightly more mixed-ancestry GWASs, we also made the following two changes:

- In the revised version, we used a different *P*-value threshold to define heterogeneous SNPs. Previously, SNPs with Q_SNP_ *P* < 5e-4 were defined as heterogeneous, whereas now the threshold was set at *P* < 5e-8. This is because Q_SNP_ is a χ2-distributed test statistic that is sensitive to sample size. As we updated our input data to the multivariate GWAS, the effective sample size increased from 92,248 to 562,507, prompting us to adjust the threshold accordingly. This new threshold also aligns with the one used by Genomic SEM developers in Grotzinger et al. (2019).
- We included possible concerns regarding mixed-ancestry into the limitation section (page 26, line 450): *‘...the contributing GWAS by Nikpay et al.[47] included mixed ancestry individuals (23%), which is cautioned against when using Genomic SEM. However, as the LD score intercepts for CAD and multimorbidity GWASs were close to 1 (0.88 and 0.99, respectively), suggests that our results are unlikely to be biased due to ancestry issues.’*

**R1.3 *r*^2^ value: An *r*^2^ of 0.6 is a very high threshold for declaring independence of signals. In these sample sizes variant- associations where the variant has an *r*^2^ of 0.5 with another variant could easily be a “shadow” of another variant. I suggest present results from *r*^2^ < 0.01. This would only affect the number of signals in the chr 9 locus and may aide interpretation of that region.**

We value the reviewer’s comment on the importance of choosing a suitable *r*^2^ cutoff to define independent genetic variants. The minimum possible *r*^2^ threshold in FUMA to define independent SNPs is *r*^2^ < 0.05, while a threshold of *r*^2^ < 0.1 is often used in similar work (e.g., Howard et al. 2019; Tcheandjieu et al., 2022). We therefore reran the analysis using both 0.05 and 0.1. Using the 0.1 threshold, we identified 11 independent SNPs, whereas with the 0.05 threshold, we identified 10 independent SNPs (i.e., only one SNP on chromosome 9 (rs3731239) was missing). To stay within the word count limit and given previous use in the literature, we decided to present only results using the *r*^2^ < 0.1 threshold in the manuscript, (pages 12-13, Table 2 and Figure 2).

**R1.4 Q heterogeneity statistic: A novel aspect of the paper is the use of the heterogeneity statistic. This Q stat helps reveal which variants could be primarily driven by single disease associations rather than be genuine multi morbidity signals. This is a key aspect of the paper and I think the Q stats should be added to table 2.**

We thank the reviewer for their positive remark. As suggested, the Q_SNP_ P-value has now been added to Table 2 (see below).

| **Locus** | **rsID** | **Chr** | **Position** | ***P*-value** | **LD SNPs** | **GWAS SNPs** | **Q_SNP_  *P*-value** | **Nearest Gene** |
| --- | --- | --- | --- | --- | --- | --- | --- | --- |
| 1 | rs10789340 | 1 | 72940273 | 3.38E-10 | 580 | 394 | **3.15e-12** | ***RPL31P12*** |
| 2 | rs9349379* | 6 | 12903957 | 3.17E-19 | 222 | 135 | **2.91e-27** | ***PHACTR1*** |
| 3 | rs10455872* | 6 | 161010118 | 4.81E-15 | 200 | 143 | **1.55e-25** | ***LPA*** |
| 3 | rs186696265* | 6 | 161111700 | 3.78E-11 | 91 | 56 | **7.53e-21** | ***RP1-81D8.3*** |
| 4 | rs2043539 | 7 | 12253880 | 1.23E-08 | 329 | 210 | **3.52e-07** | ***TMEM106B*** |
| 5 | rs3731239 | 9 | 21974218 | 3.03E-09 | 167 | 113 | **3.45e-06** | ***RP11-145E5.5: CDKN2A*** |
| 5 | rs2891168 | 9 | 22098619 | 6.88E-74 | 215 | 155 | **1.36e-27** | ***CDKN2B-AS1*** |
| 6 | rs532436 | 9 | 136149830 | 2.88E-08 | 168 | 65 | **8.93e-06** | ***ABO*** |
| 7 | rs34872471* | 10 | 114754071 | 2.32E-11 | 133 | 109 | **1.85e-17** | ***TCF7L2*** |
| 8 | rs2004910 | 12 | 121374727 | 3.60E-09 | 493 | 337 | **0.003** | ***RPL12P33*** |
| 9 | rs1962412 | 17 | 46970259 | 2.57E-08 | 510 | 359 | **0.095** | ***SUMO2P17: ATP5G1*** |

**Table 2. Independent Significant SNPs at *r*^2^ <0.1 identified in the Multivariate GWAS of Psycho-Cardiometabolic Multimorbidity.** rsID, unique identifier of independent significant single nucleotide polymorphisms (SNPs); Chr, chromosome; Position, position on hg19. LD SNPs = the number of SNPs in linkage disequilibrium (LD) with the corresponding independent significant SNP. This includes non-GWAS-tagged SNPs extracted from 1000G reference panel. GWAS SNPs = number of multimorbidity GWAS-tagged SNPs in LD (*r*^2^ < 0.1) with the corresponding independent significant SNP filtered by *P* ≤ 0.05. Q_SNP_ *P*-value = test for violation of the null hypothesis that the SNP acts entirely through the common factor. An asterisk indicates heterogeneous SNPs with a Q_SNP_ *P* < 5e-8 and directionally discordant univariate betas. While another two SNPs had Q_SNP_ *P* < 5e-8 indicative of heterogeneity, their univariate beta estimates were directionally concordant.

**Minor points:**

**R1.5 Closest/nearest gene: Although we cannot assume the closest gene is causal, because many common GWAS signals are known by local gene names, such as CDKN2 in the Chr 9 locus, it would be helpful to add closest/nearest gene labels to the key results tables.**

As suggested by the reviewer, the ‘nearest gene’ labels have now been added to Table 2 (see point R1.4 above), as well as to Table S3.

**R1. 6 Wording: Suggest do not use familiar phrases such as “to get at” in the context of “to get at the shared variance” etc.**

Following the reviewer’s advice, we replaced ‘to get at’ with ‘to estimate’.

Page 7, line 72: “*To estimate the shared variance between depression, CAD, and T2D, a common factor model was specified with all three traits loading onto a latent multimorbidity factor*.”

**R1. 7 Multimorbidity definition:** **Line 346 – the standard definition of multi morbidity is living with 2 or more conditions, but the text implies all 3 ?**

The reviewer raises a very important point. While multimorbidity is often defined as the co-occurrence of two or more conditions, in this study we focussed on the co-occurrence of three health conditions that cross the physical-mental health boundary: CAD, T2D and depression. By doing so, we expanded on previous research that had already explored shared genetic variation between two disorders (e.g., depression and CAD or depression and T2D; Haljas et al., 2018; Zhang et al., 2021). Considering all three conditions at the same time is important, as genetic variants may capture psycho-cardiometabolic risk that is not necessarily specific to any two disorders. Additionally, by incorporating three traits in the analysis, we were able to explore the genetic factor structure of multimorbidity, which would not have been possible with only two traits.

To clarify this point further we added this line in the manuscript:

Page 5, line 32: ‘*Despite many studies investigating the genetic overlap between depression, cardiovascular and metabolic diseases, the latent genetic factor structure across all three diseases has not been explored*.’

**Reviewer #2**

**R2.1 Baltramonaityte et al have performed a genome-wide association study of psycho-cardiometabolic multimorbidity using genomic SEM that is well-written and easy to follow.**

We thank Reviewer 2 for their positive comment.

**R2.2 Role of BMI: BMI is known to be causally related to both depression, CAD and T2D in mendelian randomization studies, yet not mentioned in the paper as a possible reason for this co-morbidity. I find this a bit odd and wonder about the rational for not including such a major determinant in either the introduction or the analysis. At the very least the reasoning behind this decision should be discussed.**

In response to Reviewer 2 (and 3) we updated the introduction to include a brief overview of shared life style and biological risk factors.

Page 4, line 14: ‘*The relationship between depression, CAD and T2D may be attributed in part to shared lifestyle and other risk factors such as lack of physical activity [12,13], unhealthy diet [14,15], body mass index (BMI) [16–18], altered hypothalamic-pituitary-adrenal axis [19,20], inflammation [21–23] and childhood trauma [24]. For instance, BMI and inflammation have been causally linked to all three disorders in Mendelian randomization studies [25–30], albeit with some conflicting findings [31–33].’*

Following the Reviewer’s suggestion here and also in response to the Reviewer’s comment R2.5 below, we now also include BMI and other biological risk factors (e.g., C-reactive protein) in genetic correlation analyses with multimorbidity. Please see our detailed response to point R2.5 and also Table S12.

**R2.3 Scripts: I think it would be useful if the authors provided their genomic SEM script in the supplementary.**

This is an excellent suggestion. We have now made Genomic SEM scripts available on GitHub ([*https://github.com/VilteBaltra/Psycho-cardiometabolic-multimorbidity*](https://github.com/VilteBaltra/Psycho-cardiometabolic-multimorbidity)) and refer to this in the main manuscript.

Page 26, line 506: ‘*Code underlying our analyses can be found on GitHub: https://github.com/VilteBaltra/Psycho-cardiometabolic-multimorbidity.’*

**R2.4. UKBB phenotype information: Reference UKBB phenotype information in supplementary text in main text.**

We provide extensive UKBB phenotype information in S1 Appendix methos section, which we now refer to in the main manuscript.

Page 10, line 133: ‘*[...] we first repeated the multivariate GWAS using summary statistics of European ancestry that do not include the UKBB cohort (Table 1). Subsequently, a PRS was calculated using PRSice-2 [57] to assess its association with phenotypic multimorbidity. Summary statistics for multimorbidity (excluding UKBB) provided the allelic weightings for each SNP, which were used to generate polygenic risk scores for 306,734 individuals in the UKBB cohort – our independent target sample – adjusting for 10 genetic principal components (PCs), sex and age. Multimorbidity was defined as an ordinal variable, where 0 = no disease, 1 = any one disease, 2 = any two diseases, and 3 = all three diseases (i.e., depression, CAD and T2D). See methods section in S1 Appendix for further details*.’

**R2.5 MR/LDSC with other traits, exposures and risk factors: The manuscript could potentially be made more interesting by trying to elude the causal effect of this psycho-cardiometabolic multimorbidity, e.g. mendelian randomization to find exposures causally associated with the multimorbidity or run LD score regression with the multimorbidity summary statistics to test which other traits it has a genetic correlation with.**

We fully agree with Reviewer 2 on this point, which was also raised by Reviewer 3 (R3.4). Following this suggestion, we have now investigated genetic correlations and potentially causal effects with 18 different risk factors. Results are now included in the main manuscript, S12-S14 Tables, S7-S24 Figures, and supplementary results in S1 Appendix.

Regarding genetic correlations, we added the following:

Page 19, line 331: ‘*Results from LD score regression revealed significant genetic correlations with multimorbidity for 17 out of 18 risk factors (all P_Bon_ < 0.002). The strongest genetic correlations were observed with BMI (r_g_ = 0.60, SE = 0.02, P_Bon_ = 9e-251), body fat percentage (r_g_ = 0.56, SE = 0.02, P_Bon_ = 3e-174), and C-reactive protein (CRP; r_g_ = 0.41, SE = 0.04, P_Bon_ = 3e-20). Interestingly, moderate correlations were also observed with insomnia (r_g_ = 0.36, SE = 0.03, P_Bon_ = 2e-44), neuroticism (r_g_ = 0.33, SE = 0.02, P_Bon_ = 2e-55), and childhood maltreatment (r_g_ = 0.33, SE = 0.03, P_Bon_ = 1e-32) (S12 Table)*.’

| **Phenotype** | ***r****_g_* | ***SE*** | ***P*-value** | ***P*-adjusted** |
| --- | --- | --- | --- | --- |
| Insomnia | 0.360 | 0.025 | 1.23E-45 | 2.21E-44 |
| Intelligence | -0.168 | 0.018 | 3.32E-20 | 5.98E-19 |
| Childhood maltreatment | 0.332 | 0.027 | 7.11E-34 | 1.28E-32 |
| Neuroticism | 0.330 | 0.021 | 1.33E-56 | 2.40E-55 |
| SESA | 0.208 | 0.023 | 9.08E-20 | 1.63E-18 |
| Worry | 0.182 | 0.023 | 6.73E-16 | 1.21E-14 |
| Income (MTAG) | -0.345 | 0.019 | 2.77E-75 | 4.99E-74 |
| Current smoking | 0.338 | 0.023 | 1.41E-49 | 2.54E-48 |
| BMI | 0.597 | 0.018 | 5.03E-252 | 9.05E-251 |
| Body fat percentage | 0.567 | 0.020 | 1.72E-175 | 3.10E-174 |
| Systolic blood pressure | 0.226 | 0.018 | 3.30E-35 | 5.93E-34 |
| Diastolic blood pressure | 0.169 | 0.018 | 2.31E-20 | 4.15E-19 |
| C-reactive protein | 0.412 | 0.043 | 1.53E-21 | 2.76E-20 |
| Fasting insulin | 0.350 | 0.051 | 4.71E-12 | 8.47E-11 |
| LDL cholesterol | 0.132 | 0.034 | 9.34E-05 | 0.0017 |
| HDL cholesterol | -0.356 | 0.027 | 2.34E-40 | 4.22E-39 |
| Total cholesterol | 0.089 | 0.031 | 0.0036 | 0.0648 |
| Triglycerides | 0.362 | 0.041 | 1.04E-18 | 1.88E-17 |
| Coronary artery disease* | 0.525 | 0.030 | 5.62E-71 | 1.01E-69 |
| Type 2 diabetes* | 0.897 | 0.006 | 0.00+00 | 0.00+00 |
| Major depression* | 0.489 | 0.018 | 1.24E-166 | 2.23E-165 |

**Abbreviated S12 Table. Results of LD Score regression analyses between psycho-cardiometabolic multimorbidity and various risk factors (for full table, see S12 Table in the supplementary materials).** *Contributing traits to multimorbidity GWAS. LD = linkage disequilibrium, *SE* = standard error, *P*-adjusted = Bonferroni adjusted *P*-values, *r_g_*, genetic correlation. SESA = sensitivity to environmental stress and adversity, MTAG = multi-trait analysis of GWAS.

Regarding Mendelian randomization, we added this:

Page 19, line 338: ‘*Mendelian randomization analyses revealed potentially causal associations of BMI, body fat percentage, LDL cholesterol, total cholesterol, fasting insulin, income, insomnia, and childhood maltreatment that survived correction for multiple testing using the Benjamini-Hochberg (BH) false discovery rate. Sensitivity analyses estimates using MR-Egger, simple mode, weighted median and weighted mode methods were generally consistent for all these traits (with a minimum of three out of four sensitivity analyses having significant causal estimates), indicating robustness of our primary results (Fig 4; S13 Table). Evidence for neuroticism, blood pressure traits, and triglycerides was more mixed. We found little or no evidence to support a causal effect of intelligence, worry, sensitivity to environmental stress and adversity, HDL cholesterol, smoking status, and C-reactive protein (S7-S24 Fig; S13 Table). Results from the remaining sensitivity analyses and MRlap are reported in the supplementary material (S1 Appendix, results section; S7-24 Fig; S13-S14 Table).’*

We also briefly refer to these findings in our discussion section:

Page 23, line 440: ‘*This is further supported by genetic correlation and MR results between a number of depression-related risk factors, such as insomnia, childhood maltreatment and adiposity traits.*

*Overall, while we observed weak-to-strong genetic correlations between 17 risk factors and multimorbidity, only seven of these associations (BMI, body fat percentage, LDL cholesterol, total cholesterol, fasting insulin, income, and insomnia) demonstrated consistent estimates across most MR analyses, suggesting potentially causal effects.’*

**Reviewer #3**

**R3.1 Rationale: The authors used genome wide association studies of coronary heart disease, T2D and depression to investigate the shared genetic factors between these three conditions.**

**The introduction is unconvincing in why the authors decided to include T2D and link this to depression is unclear, biological mechanisms were not explained.**

We thank the Reviewer for pointing this out and would like to refer to our response to Reviewer 2 (R2.2) where we addressed a similar comment. Specifically, our introduction now highlights inflammation and alterations in hypothalamic-pituitary-adrenal axis as potential biological mechanisms underlying these conditions.

Page 4, line 14: ‘*The relationship between depression, CAD and T2D may be attributed in part to shared lifestyle and other risk factors such as lack of physical activity [12,13], unhealthy diet [14,15], body mass index (BMI) [16–18], altered hypothalamic-pituitary-adrenal axis [19,20], inflammation [21–23] and childhood trauma [24]. For instance, BMI and inflammation have been causally linked to all three disorders in Mendelian randomization studies [25–30], albeit with some conflicting findings [31–33].’*

**R3.2 CAD GWAS: The authors did not use the latest CKD GWAS. They used one published in 2011 so quite outdated and with a much fewer number of individuals and significant snps. Without the use of the latest GWAS the publication of the paper will be already outdated so I suggest they re-do it.**

We agree with the Reviewer’s comment and have re-run the analyses using a more recent GWAS, as described in detail in our response to Reviewer 1 (R1.2).

To briefly summarize, following recommendations by both Reviewer 1 and 3, we decided to rerun the analyses in the following two ways:

- include the largest GWAS for each trait for maximum power (GWAS_with.UKBB_)
- include the largest GWAS for each trait *excluding* UK Biobank data (GWAS_without.UKBB_)

The genetic correlations between our original multimorbidity GWAS results (using Schunkert et al., 2011), GWAS_with.UKBB_, and GWAS_without.UKBB_ were very high, suggesting that all versions were capturing a similar genetic signal. To capitalize on power as suggested by Reviewer 1 and 3, we decided to prioritize GWAS_with.UKBB_ in the manuscript and use GWAS_without.UKBB_ for the follow-up PRS analyses. Our updated results confirmed our original findings and improved in precision. Please see our response to R1.2 for further details.

**R3.3 Depression diagnosis versus symptoms: I would expect that the sensitivity analysis would only include cohorts where depression diagnosis was properly validated using structured clinical questionnaire, and not including only those with depressive symptoms. However this important point for this condition in particular was not discussed.**

We thank the reviewer for bringing up this important point.

The genome-wide meta-analysis of depression by Howard et al. (2019) included three cohorts: PGC_139k, 23andMe_307 and the UKBB. These cohorts defined case status as follows:

- PGC_139k: a lifetime diagnosis of depression determined using structured diagnostic instruments;
- 23andMe_307k: self-reported clinical diagnosis or treatment for depression by a medical professional;
- UKBB: ever self-reported help-seeking for problems with nerves, tension, anxiety, or depression (i.e., a broader depression phenotype; see S1 Table).

In line with suggestions by Reviewer 3 (and Reviewer 1, see point R1.2), our revised multimorbidity GWAS included summary-level data for depression that was based on all three of these cohorts (GWAS_with.UKBB_). However, our multivariate GWAS without UKBB (GWAS_without.UKBB_) only included summary-level data based on PGC_139k and 23andMe_307k (i.e., case status primarily based on clinical diagnosis).

We observed comparable results across both GWAS versions, with a genetic correlation of 0.98. This suggested that differentiating between clinically versus broadly defined depression phenotypes did not seem to greatly impact the results of the current genome-wide association analysis.

S1 Appendix, page 8, section 1: *‘Overall, consistent findings were observed when repeating the analysis using the largest available GWASs for each trait, but this time excluding the UKBB cohort. Briefly, we observed positive genetic correlations among each pair of traits (r_g CAD-T2D_ = 0.41, SE = 0.04, P = 1e-20; r_g CAD-MD_ = 0.10, SE = 0.3, P = .002); r_g T2D-MD_ = 0.10, SE = 0.03, P = .002), which aligned closely in terms of their magnitude with the original genetic correlations with UKBB. The factor structure was also comparable, with the common factor explaining the greatest proportion of variance in T2D (R^2^ = 0.42) and CAD (R^2^ = 0.40), followed by depression (R^2^ = 0.03). The multivariate GWAS identified 1,312 genome-wide significant SNPs, of which 30 were independent SNPs (S4 Table). Summary statistics for multimorbidity without UKBB correlated very strongly with summary statistics with UKBB (r_g_ = 0.98, SE = 0.01, P < .001).’*

This finding is also in line with the strong genetic correlations between all three contributing GWAS datasets (*r*g = 0.85-0.87) reported by Howard et al. (2019), despite variations in how the phenotype was defined.

**R3.4 Risk factors: The authors did not investigate any cardiovascular, diabetes or depression risk factors.**

Please see our detailed response to Reviewer 2 (R2.5), who raised a similar concern.

To briefly summarize, we have now investigated genetic correlations with 18 different risk factors. LD score regression showed significant genetic correlations with multimorbidity for 17 out of 18 risk factors. Mendelian randomization analyses revealed generally consistent causal associations for seven of these traits (BMI, body fat percentage, LDL cholesterol, total cholesterol, fasting insulin, income and insomnia) on multimorbidity across primary and sensitivity analyses. Results are now included in the main manuscript, S12-S14 Tables, S7-S24 Figures, and supplementary results in S1 Appendix. A more detailed description is provided in our response to R2.5.

**R3.5 Heterogeneity: The authors did not investigate heterogeneity.**

We report genetic heterogeneity statistics throughout the results section, which indicate the extent to which the SNP effect deviates from the common factor structure, with smaller Q_SNP_ *P*-values suggesting greater heterogeneity (Table 2, Fig 2A, and S4 Table).

We also evaluate heterogeneity of independent significant SNPs in our discussion section:

Page 13, line 204: ‘*An inspection of the univariate betas within the three contributing GWASs revealed directionally discordant estimates across the three traits for four of the 11 independent SNPs (S5 Table), indicating that these particular SNPs were unlikely to operate via the common factor but, instead, were more likely to be phenotype specific. The remaining seven SNPs were consistent in the direction of their univariate betas, with four SNPs showing the largest effects for CAD and T2D, and three SNPs having comparable estimates across the three traits (for regional plots of these SNPs see Fig 2).*’

**R3.6 Functional analysis: There were no functional analysis of the shared loci.**

We report functional analysis of multimorbidity in the following sections of our Results:

- Functional annotation and gene mapping (Page 14, line 240)
- Gene-based analysis of all SNPs (Page 15, line 248)
- Pathway enrichment analysis and tissue specificity of prioritised genes (Page 16, line 264)
- MAGMA gene-set and tissue expression analyses (Page 16, line 277)

Additionally, we now:

- include ‘nearest gene’ labels in Table 2 and Table S3 (see point R1.4 above)
- include Table 3, listing 18 genes identified using four distinct methods: MAGMA gene-based analysis, positional, eQTL and chromatin interaction mapping
- further expand our discussion of these results in the main manuscript (see below).

| **Gene** | **Status** | **Chr** | **Start** | **End** | **nSNPs** | **Z** | ***P*-value^a^** |
| --- | --- | --- | --- | --- | --- | --- | --- |
| *NEGR1* | confirmed | 1 | 71851623 | 72758417 | 1673 | 5.37 | 3.87E-08 |
| *TMEM106B* | confirmed | 7 | 12240867 | 12292993 | 272 | 5.40 | 3.30E-08 |
| *RP11-145E5.5* | novel | 9 | 21792635 | 22042985 | 412 | 6.91 | 2.40E-12 |
| *C9orf53* | novel | 9 | 21957137 | 21977738 | 19 | 6.07 | 6.34E-10 |
| *CDKN2A* | novel | 9 | 21957751 | 22005300 | 48 | 7.08 | 7.40E-13 |
| *CDKN2B* | novel | 9 | 21992902 | 22019362 | 37 | 7.85 | 2.05E-15 |
| *SPPL3* | novel | 12 | 121190313 | 121352174 | 463 | 5.78 | 3.70E-09 |
| *AC079602.1* | novel | 12 | 121397641 | 121420095 | 66 | 6.27 | 1.85E-10 |
| *HNF1A* | confirmed | 12 | 121406346 | 121450315 | 143 | 5.87 | 2.18E-09 |
| *C12orf43* | novel | 12 | 121430225 | 121464305 | 110 | 5.74 | 4.63E-09 |
| *OASL* | novel | 12 | 121448095 | 121487045 | 118 | 5.75 | 4.42E-09 |
| *TTLL6* | novel | 17 | 46829597 | 46904576 | 205 | 5.33 | 4.99E-08 |
| *ATP5G1* | novel | 17 | 46960127 | 46983233 | 48 | 5.66 | 7.58E-09 |
| *UBE2Z* | novel | 17 | 46975731 | 47016418 | 112 | 5.43 | 2.82E-08 |
| *SNF8* | novel | 17 | 46996678 | 47032479 | 110 | 5.47 | 2.19E-08 |
| *GIP* | novel | 17 | 47025916 | 47055958 | 92 | 5.62 | 9.51E-09 |
| *IGF2BP1* | novel | 17 | 47064774 | 47143012 | 125 | 5.30 | 5.78E-08 |
| *TCF4* | novel | 18 | 52879562 | 53342018 | 668 | 4.75 | 1.03E-06 |

**Table 3. MAGMA results for 18 genes identified using four distinct methods: MAGMA gene-based analysis, positional, eQTL and chromatin interaction mapping.** Confirmed status refers to genes that have been associated with all three disorders (i.e., coronary artery disease, type 2 diabetes and depression) in previous studies. Novel status refers to genes that are for the first time being linked to all three disorders. Chr, chromosome; nSNPs, number of single nucleotide polymorphisms; Z, Z-statistic; eQTL, expression quantitative trait loci.

^a^Bonferroni corrected *P*-value threshold was set at 0.05 / (the number of tested genes) = 2.64e-6.

Page 21, line 391: *‘Focusing on non-heterogeneous SNPs to minimize genetic signals driven by a single disorder, FUMA prioritised 200 genes putatively associated with multimorbidity, 18 of which were also identified using positional mapping, eQTL mapping, chromatin interaction mapping, and MAGMA gene-based analysis, thereby providing the most consistent support for these genes. Three of these genes (NEGR1, TMEM106B, HNF1A) have been linked to each of the three diseases (CAD, T2D and depression) in previous studies [44,65–74], supporting their probable involvement in psycho-cardiometabolic multimorbidity. The remaining 15 were completely novel (e.g., SNF8 and AC079602.1) or had only been linked to one or two diseases. For example, while SPPL3 and TCF4 have been associated with T2D [42,67] and depression [72,75,76] (as well as various other psychiatric traits [77,78]), an association with CAD has not yet been reported. However, SPPL3 and TCF4 have been linked to CAD risk factors such as cholesterol levels [79,80] and CRP [79,81,82], suggesting a potential role in multimorbidity. Similarly, UBE2Z, GIP and IGF2BP1 genes have been linked to CAD [70,83,84], T2D [66,67,85], and depression-related traits such as insomnia, BMI, educational attainment, CRP levels, platelet count and smoking [82,86–92]. As such, even though a direct association with depression has not yet been established, their relevance in psycho-cardiometabolic multimorbidity seems biologically plausible.*

*A similar pattern was observed for the other genes, whereby the majority tagged common risk factors for CAD, T2D and depression, such as adiposity related traits (BMI, body fat percentage, waist/hip circumference), inflammatory markers (CRP, interleukin-6, interleukin-5), lipids (low- and high-density lipoprotein levels), platelet traits (platelet count, plateletcrit), and N-glycan levels [42,70,79,87,93–100]. Previous knowledge for three of the identified genes (RP11-145E5.5, SNF8, AC079602.1) was weak, suggesting potentially novel targets for follow-up in relation to multimorbidity.’*

Page 23, line 432: *‘Tissue specificity analysis indicated increased gene expression relative to other tissue types in the cerebellum and the pituitary gland. This is of interest as the structure and function of these regions seems to be altered in depression. For example, individuals with depression tend to have an overactive hypothalamic-pituitary-adrenal axis (our main stress response system), leading to increased cortisol levels and suppressed immune responses [20]. Similarly, with regard to the cerebellum, important cerebellar alterations have been identified in patients with depression [111], impacting emotion regulation ability [112].’*

**R3.7 Gender: The authors did not separate the findings by gender which would be important in this context.**

In response to this suggestion, we now report results separately for males and females in the main manuscript and Tables S10 and S11.

Page 19, line 324: *‘A sex-stratified analysis using multinomial logistic regression revealed comparable results for males and females. Specifically, relative to the healthy group, for every one standard deviation increase in multimorbidity-PRS, the odds of experiencing multimorbidity increased by 94% (OR = 1.94, 95% CI = 1.73–2.17) for males and 86% (OR = 1.86, 95% CI = 1.57–2.20) for females (S10 and S11 Table).’*

| **Outcome** | **PRS all sample**  adjusted OR (95% CI) | **PRS males**  adjusted OR (95% CI) | **PRS females**  adjusted OR (95% CI) |
| --- | --- | --- | --- |
| Healthy | Ref | Ref | Ref |
| MD only | 1.07 (1.06-1.08) | 1.07 (1.05-1.09) | 1.07 (1.05-1.08) |
| T2D only | 1.36 (1.33-1.38) | 1.36 (1.32-1.39) | 1.36 (1.32-1.40) |
| CAD only | 1.38 (1.35-1.41) | 1.41 (1.38-1.45) | 1.29 (1.25-1.34) |
| MD and T2D | 1.46 (1.40-1.52) | 1.45 (1.36-1.54) | 1.47 (1.39-1.56) |
| MD and CAD | 1.46 (1.39-1.53) | 1.48 (1.38-1.57) | 1.43 (1.33-1.55) |
| CAD and T2D | 1.73 (1.66-1.81) | 1.76 (1.67-1.85) | 1.64 (1.49-1.80) |
| Multimorbidity | 1.91 (1.74-2.10) | 1.94 (1.73-2.17) | 1.86 (1.57-2.20) |

**S11 Table. Sex-stratified results for the association between multimorbidity polygenic risk score and disease status in UK Biobank.** Results from multinomial logistic regression adjusted for age and sex (*N*_all_ = 306,734, *N*_males_ = 141,720, *N*_females_ = 165,014). Polygenic risk score has been standardised. Reference category = healthy. Multimorbidity = CAD + T2D + MD. OR, odds ratio; CI, confidence interval; MM, multimorbidity; CAD, coronary artery disease; T2D, type 2 diabetes; MD, major depression.

**R3.8 Previous study: The paper is in a way very similar idea to the already published article in PLOS Genetics 2022 Shared genetic loci between depression and cardio metabolic traits, but of much lower quality.**

We thank the Reviewer for bringing this paper to our attention, which we now cite in our manuscript on page 5, line 33.

Torgersen et al. (2022) used the bivariate causal mixture model (MiXeR) and the conditional/conjunctional false discovery rate (pleioFDR) methods to quantify genome-wide overlap and to identify shared loci between depression, coronary artery disease, and cardiovascular disease risk factors. After careful consideration of this paper, we would like to highlight that our studies differed in the following important ways:

1. **Distinct methods**: we employed Genomic structural equation modelling, which
   a. enabled us to model the shared genetic architecture across depression, CAD and T2D, with a latent multimorbidity factor representing broad genetic liability
   b. boosted power for cross-trait SNP discovery

This contrasted Torgersen et al. approach who quantified genome-wide overlap using a broader range of phenotypes: depression, CAD and nine cardiovascular risk factors and used pleioFDR to directly identify shared genetic loci (i.e., without first modelling their genetic factor structure).

2. **PRS**: we validated our latent multimorbidity factor by testing associations between polygenic risk scores for multimorbidity and disease status in the UKBB cohort (*N* = 306,734)
3. **Genetic correlations**: we assessed genetic correlations between multimorbidity and 18 risk factors (see R2.5)
4. **MR**: we explored causal relationships with these risk factors using Mendelian randomization (see R2.5).

Building on studies such as Torgersen et al. (2022), our study adds novel insights to common genetic pathways of multimorbidity by characterizing the genetic factor structure of multimorbidity, providing full summary statistics for SNP associations with the latent multimorbidity factor, and identifying potentially causal risk factors. We therefore strongly believe that these findings make a valuable contribution to multimorbidity research and thank the reviewers for significantly strengthening the manuscript.

**References**

Haljas, K., Amare, A. T., Alizadeh, B. Z., Hsu, Y.-H., Mosley, T., Newman, A., Murabito, J., Tiemeier, H., Tanaka, T., van Duijn, C., Ding, J., Llewellyn, D. J., Bennett, D. A., Terracciano, A., Launer, L., Ladwig, K.-H., Cornelis, M. C., Teumer, A., Grabe, H., … Lahti, J. (2018). Bivariate Genome-Wide Association Study of Depressive Symptoms With Type 2 Diabetes and Quantitative Glycemic Traits: *Psychosomatic Medicine*, *80*(3), 242–251. https://doi.org/10.1097/PSY.0000000000000555

Howard, D. M., Adams, M. J., Clarke, T.-K., Hafferty, J. D., Gibson, J., Shirali, M., Coleman, J. R. I., Hagenaars, S. P., Ward, J., Wigmore, E. M., Alloza, C., Shen, X., Barbu, M. C., Xu, E. Y., Whalley, H. C., Marioni, R. E., Porteous, D. J., Davies, G., Deary, I. J., … McIntosh, A. M. (2019). Genome-wide meta-analysis of depression identifies 102 independent variants and highlights the importance of the prefrontal brain regions. *Nature Neuroscience*, *22*(3), 343–352. https://doi.org/10.1038/s41593-018-0326-7

Mahajan, A., Taliun, D., Thurner, M., Robertson, N. R., Torres, J. M., Rayner, N. W., Payne, A. J., Steinthorsdottir, V., Scott, R. A., Grarup, N., Cook, J. P., Schmidt, E. M., Wuttke, M., Sarnowski, C., Mägi, R., Nano, J., Gieger, C., Trompet, S., Lecoeur, C., … McCarthy, M. I. (2018). Fine-mapping type 2 diabetes loci to single-variant resolution using high-density imputation and islet-specific epigenome maps. *Nature Genetics*, *50*(11), Article 11. https://doi.org/10.1038/s41588-018-0241-6

Nikpay, M., Goel, A., Won, H.-H., Hall, L. M., Willenborg, C., Kanoni, S., Saleheen, D., Kyriakou, T., Nelson, C. P., Hopewell, J. C., Webb, T. R., Zeng, L., Dehghan, A., Alver, M., Armasu, S. M., Auro, K., Bjonnes, A., Chasman, D. I., Chen, S., … Farrall, M. (2015). A comprehensive 1000 Genomes-based genome-wide association meta-analysis of coronary artery disease. *Nature Genetics*, *47*(10), 1121–1130. https://doi.org/10.1038/ng.3396

Schunkert, H., König, I. R., Kathiresan, S., Reilly, M. P., Assimes, T. L., Holm, H., Preuss, M., Stewart, A. F. R., Barbalic, M., Gieger, C., Absher, D., Aherrahrou, Z., Allayee, H., Altshuler, D., Anand, S. S., Andersen, K., Anderson, J. L., Ardissino, D., Ball, S. G., … Erdmann, J. (2011). Large-scale association analysis identifies 13 new susceptibility loci for coronary artery disease. *Nature Genetics*, *43*(4), 333–338. https://doi.org/10.1038/ng.784

Scott, R. A., Scott, L. J., Mägi, R., Marullo, L., Gaulton, K. J., Kaakinen, M., Pervjakova, N., Pers, T. H., Johnson, A. D., Eicher, J. D., Jackson, A. U., Ferreira, T., Lee, Y., Ma, C., Steinthorsdottir, V., Thorleifsson, G., Qi, L., Van Zuydam, N. R., Mahajan, A., … Prokopenko, I. (2017). An Expanded Genome-Wide Association Study of Type 2 Diabetes in Europeans. *Diabetes*, *66*(11), 2888–2902. https://doi.org/10.2337/db16-1253

Tcheandjieu, C., Zhu, X., Hilliard, A. T., Clarke, S. L., Napolioni, V., Ma, S., Lee, K. M., Fang, H., Chen, F., Lu, Y., Tsao, N. L., Raghavan, S., Koyama, S., Gorman, B. R., Vujkovic, M., Klarin, D., Levin, M. G., Sinnott-Armstrong, N., Wojcik, G. L., … Assimes, T. L. (2022). Large-scale genome-wide association study of coronary artery disease in genetically diverse populations. *Nature Medicine*, *28*(8), 1679–1692. https://doi.org/10.1038/s41591-022-01891-3

Torgersen, K., Rahman, Z., Bahrami, S., Hindley, G. F. L., Parker, N., Frei, O., Shadrin, A., O’Connell, K. S., Tesli, M., Smeland, O. B., Munkhaugen, J., Djurovic, S., Dammen, T., & Andreassen, O. A. (2022). Shared genetic loci between depression and cardiometabolic traits. *PLOS Genetics*, *18*(5), e1010161. https://doi.org/10.1371/journal.pgen.1010161

Zhang, F., Cao, H., & Baranova, A. (2021). Shared Genetic Liability and Causal Associations Between Major Depressive Disorder and Cardiovascular Diseases. *Frontiers in Cardiovascular Medicine*, *8*, 735136. https://doi.org/10.3389/fcvm.2021.735136
