# Supplementary material for: A multivariate genome-wide association study of psycho-cardiometabolic multimorbidity
Source: PLoS Genet. 2023 Jun 30;19(6):e1010508. doi: 10.1371/journal.pgen.1010508 (PMC10343069; doi:10.1371/journal.pgen.1010508)
Supplement: Attachment — Submitted filename: Reviewer-response-round2-20230531.docx [file pgen.1010508.s019.docx]

**Response to Reviewer’s Comments**

We thank the Reviewer for their comment and suggestion. The Reviewer’s comment is in bold. Our response is in regular font (in blue). In *italics* we include the updated excerpts from the manuscript.

**Reviewer #1:**

**R1.1 Limitation section: Many thanks for doing such a thorough job answering my queries. My only final comment is that the discussion limitation section ideally needs to be a little more circumspect about how well the latent factor is genuinely representing MM or just the individual conditions. Ten of the 11 variants in the main table reach p<0.05 and most are p<5x10-8 for their heterogeneity values, indicating they are primarily driven by one trait. The use of 5x10-8 as the QSNP threshold is fine but could still mean you have a lot of enrichment for heterogeneous variants, making all the downstream analysis biased towards finding pathways affecting one trait. A quick QQ plot of the QSNPs could enlighten this issue?**

This is an important point, and we thank the reviewer for highlighting it.

We removed heterogeneous variants (heterogeneity Q_SNP_ *P* < 5e−8 and directionally discordant univariate effect estimates) from all our downstream analyses, but, as the reviewer correctly highlighted, there was suggestive evidence of heterogeneity for multiple SNPs with Q_SNP_ *P* < 0.05 (see Figure S3 for a Q-Q plot of the Q_SNP_ *P*-values, which suggest a larger degree of heterogeneity than expected under the null hypothesis). As stated by the Reviewer, this may indeed indicate that our results are partly biased towards pathways related to one or two traits. To gain further insight into this issue, we correlated the heterogeneity *Q*_SNP_ *P*-values with the GWAS *P*-values. We observed a minimal correlation (*r* = 0.055) between these two sets of *P*-values, suggesting that smaller heterogeneity *P*-values do not necessarily imply smaller GWAS *P*-values. In other words, despite the presence of residual heterogeneity, associations between the genetic variants and multimorbidity seemed to be largely independent of heterogeneity.

**S3 Fig. A quantile-quantile plot of heterogeneity Q_SNP_ *P*-values for the multivariate GWAS of psycho-cardiometabolic multimorbidity.** *The observed –log10(P) -values in black are plotted against their expected values under the null hypothesis (red line). To assess a possible inflation of heterogeneity for multimorbidity-associated SNPs (rather than SNPs tagging one or two of the constituting diseases), variants with a heterogeneity Q_SNP_ P < 5e-8 and directionally discordant univariate effect estimates were removed prior to plotting this data. SNPs = single nucleotide polymorphisms.*

However, we overall agree with the reviewer’s concern, despite these reassuring findings, and updated the discussion section to include the following:

Page 24, line 454: ‘*Third, although we removed SNPs with strong evidence for heterogeneity (Q_SNP_ P < 5e−8 and* *directionally discordant univariate effect estimates), there were still many variants left in the analyses with suggestive evidence for heterogeneity. This means that our downstream analyses may be biased towards pathways related to any one or two constituting diseases. Therefore, when using multimorbidity summary statistics in future studies, it may be appropriate to apply an even more stringent heterogeneity threshold (e.g., P < 5e-6), depending on the nature of the investigation*.’

We also included the following guidance in Bath’s Research Data Archive where summary statistics for multimorbidity are deposited (to preview the dataset enter the following access token ‘2VHzALOllpjFcHcyWV52zKP59XzxbjBg’ here

<https://researchdata.bath.ac.uk/preview/1179>):

‘*When using multimorbidity-associated variants as genetic instruments in Mendelian randomization analysis we recommend to only use non-heterogeneous SNPs. At the minimum, we recommend removing variants with Q_SNP_ P < 5e-8 and directionally discordant univariate effect estimates (option 1 below), as done in the non-heterogenous summary statistics versions (PCM_multimorbidity_summary_stats_noUKBB_non-het.txt.gz and*

*PCM_multimorbidity_summary_stats_withUKBB_non-het.txt.gz). To ensure your analysis captures multimorbidity between coronary artery disease, type 2 diabetes and major depression, you may also apply a more stringent threshold for removing heterogeneous variants (e.g., Q_SNP_ P < 5e-6; option 2 below). A sample code for implementing this in R is included below:*

*# load tidyverse package*

*library(tidyverse)*

*# read in summary statistics*

*sumstats <- read.delim("PCM_multimorbidity_summary_stats_withUKBB_non-het.txt.gz")*

*# Option 1: remove discordant variants with Q_SNP_ P < 5e-8*

*data_not_concordant <- sumstats %>% filter(concordant == "FALSE")*

*mm_snp_remove <- sumstats %>% filter(SNP %in% data_not_concordant$SNP) %>% filter(chisq_pval < 0.00000005)*

*sumstas_nonhet <- sumstas %>% filter(!SNP %in% mm_snp_remove$SNP)*

*# save file*

*write.table(sumstas_nonhet, file = “newfile.txt", quote =F, sep = "\t", row.names = F)*

*# Option 2: remove variants with Q_SNP_ P < 5e-6*

*sumstas_nonhet <- sumstas %>% filter(chisq_pval < 0.000005)*

*# save file*

*write.table(sumstas_nonhet, file = “newfile.txt", quote =F, sep = "\t", row.names = F)’*

**Additional comment from the authors:** Please note that we also corrected a minor omission in the abstract: ‘Childhood maltreatment’ and ‘income’ were identified as two of eight potentially causal risk factors (e.g., SM Table 13), but not mentioned in the abstract. We added these two factors to the abstract, and highlighted this change in blue in the submitted, revised manuscript, which now reads as follows: “*Mendelian randomization analyses suggested potentially causal effects of BMI, body fat percentage, LDL cholesterol, total cholesterol, fasting insulin, income, insomnia, and childhood maltreatment.”*
